# PLAUR^+^ Neutrophils Drive Anti‐PD‐1 Therapy Resistance in Patients with Hepatocellular Carcinoma by Shaping an Immunosuppressive Microenvironment

**DOI:** 10.1002/advs.202507167

**Published:** 2025-07-17

**Authors:** Shaoqing Liu, Yanzhao Zhou, Gaoxiang Li, Bingwen Zhu, Fang Wu, Jinxue Zhou, Xiaobing Chen, Bo Qin, Yanxia Gao, Fazhan Wang, Yong Jiang, Wenxin Xu

**Affiliations:** ^1^ Department of Breast Surgery Translational Medical Center The First Affiliated Hospital of Zhengzhou University Zhengzhou 450001 China; ^2^ State Key Laboratory of Metabolic Dysregulation & Prevention and Treatment of Esophageal Cancer Henan International Joint Laboratory of Infection and Immunity Henan Key Laboratory of Critical Care Medicine Department of Emergency Medicine The First Affiliated Hospital of Zhengzhou University Zhengzhou 450001 China; ^3^ Department of Medical Oncology The Affiliated Cancer Hospital of Zhengzhou University & Henan Cancer Hospital Zhengzhou 450008 China; ^4^ State Key Laboratory of Systems Medicine for Cancer Division of Cardiology Renji Hospital School of Medicine Shanghai Jiao Tong University Shanghai 200127 China; ^5^ Institute of Infection and Immunity Henan Academy of Innovations in Medical Science Zhengzhou 451163 China; ^6^ Department of Immunology School of Basic Medical Sciences Zhengzhou University Zhengzhou 450001 China; ^7^ Department of Hepatobiliary and Pancreatic Surgery The Affiliated Cancer Hospital of Zhengzhou University & Henan Cancer Hospital Zhengzhou 450008 China; ^8^ Medical Research Center The First Affiliated Hospital of Zhengzhou University Zhengzhou University Zhengzhou Henan 450052 China; ^9^ Department of Liver Surgery and Transplantation Liver Cancer Institute Zhongshan Hospital Fudan University Key Laboratory of Carcinogenesis and Cancer Invasion Ministry of Education Shanghai 200032 P. R. China

**Keywords:** biomarker, hepatocellular carcinoma, immunotherapy, neutrophil, urokinase‐type plasminogen activator receptor

## Abstract

Hepatocellular carcinoma (HCC) is characterized by an immunosuppressive tumor microenvironment (TME) that limits the efficacy of immune checkpoint inhibitors. However, the role of distinct neutrophil subsets within the TME in mediating tumor resistance to immunotherapy remains poorly understood. Here, the present study reveals that the urokinase‐type plasminogen activator receptor (PLAUR)^+^ neutrophils are enriched in immunotherapy non‐responders and correlate with poor prognosis. Through multi‐omics analyses of clinical cohorts and preclinical models, PLAUR^+^ neutrophils identify as a pivotal driver of immunotherapy resistance by shaping an immunosuppressive TME. Mechanistically, spatial transcriptomics and single‐cell RNA sequencing reveal that PLAUR^+^ neutrophils orchestrate immune evasion by CD8^+^ T cell exclusion and macrophage‐dependent immune suppression. Through the structure‐based virtual screening, a novel PLAUR inhibitor is identified that could reverse the immunosuppressive phenotype of neutrophils. In various in vivo tumor models, PLAUR inhibitor suppresses tumor growth and potentiates the efficacy of anti‐programmed cell death protein 1 (PD‐1) therapy. These results demonstrate that PLAUR^+^ neutrophils serve as a critical regulator of immunotherapy resistance and targeting PLAUR is a promising strategy to augment the efficacy of anti‐PD‐1 therapy in HCC.

## Graphical abstract

The present study reveals that PLAUR^+^ neutrophils are enriched in immunotherapy non‐responders and correlate with poor prognosis. PLAUR^+^ neutrophils dictate immunotherapy resistance in hepatocellular carcinoma by forming an immunosuppressive tumor microenvironment characterized by CD8^+^ T cell exclusion and macrophage‐dependent immune suppression. The development of DAPTA, a novel PLAUR inhibitor, represents a promising therapeutic strategy to improve anti‐PD‐1 therapy efficacy.

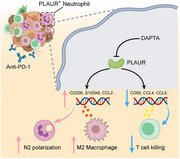

## 1 Introduction

Hepatocellular carcinoma (HCC) represents a major global health burden, accounting for approximately 90% of primary liver cancers and ranking as the third leading cause of cancer‐related mortality worldwide.^[^ ^1^ ^]^ Immune checkpoint inhibitors (ICIs) have emerged as a novel therapeutic strategy for unresectable HCC, offering durable responses in a subset of patients.^[^ ^2^ ^]^ However, the objective response rate of ICIs in HCC remains limited to approximately 15%–20% due to the high heterogeneity and aggressiveness of HCC.^[^ ^3^, ^4^ ^]^ Therefore, it is imperative to elucidate the mechanisms underlying immunotherapy resistance and identify potential targets to augment therapeutic efficacy.

The tumor microenvironment (TME) of HCC is increasingly recognized as a critical factor influencing immunotherapy efficacy.^[^ ^5^, ^6^ ^]^ Characterized by profound immunosuppression, the HCC TME is enriched with cancer‐associated fibroblasts (CAFs), tumor‐associated macrophages (TAMs), and tumor‐associated neutrophils (TANs), which collectively establish barriers to T cell infiltration and function.^[^ ^7^, ^8^, ^9^ ^]^ Among these, TANs exhibited distinct polarization states in the TME: an antitumor “N1” phenotype characterized by cytotoxic activity and an immunosuppressive “N2” phenotype that promotes angiogenesis, extracellular matrix remodeling, and T cell dysfunction.^[^ ^10^, ^11^ ^]^ Despite these insights, the functional heterogeneity of neutrophils in HCC and their contribution to ICIs resistance remain poorly understood, limiting the development of targeted therapeutic strategies.

The urokinase‐type plasminogen activator receptor (PLAUR, uPAR) belongs to the plasminogen activation system, which consists of three structural domains attached to cell membranes via glycolipid anchors. Previous studies had revealed that PLAUR expression is upregulated in various tumors and correlates with poor prognosis.^[^ ^12^, ^13^, ^14^, ^15^ ^]^ In addition, PLAUR has been implicated in inducing glycolysis and oxidative phosphorylation reprogramming in tumor cells.^[^ ^16^ ^]^ However, previous studies on PLAUR have mostly focused on tumor cells, and the expression profile and major role of PLAUR in the TME of HCC remain to be further elucidated.

Based on multi‐omics analyses, the present study revealed that PLAUR^+^ neutrophils serve as pivotal regulators of anti‐programmed death‐1 (PD‐1) therapy resistance in HCC by orchestrating TAMs‐driven immunosuppression and CD8^+^ T cell exclusion. Moreover, through high‐throughput virtual screening, we discovered a novel PLAUR inhibitor that exhibited potent antitumor efficacy and sensitized anti‐PD‐1 therapy. These findings provide a novel illustration of the immunological significance and potential therapeutic implications of PLAUR^+^ neutrophils in HCC.

## 2 Results

### 2.1 PLAUR^+^ Neutrophils Are Correlated with the Resistance to Anti‐PD‐1 Therapy and Poor Prognosis for HCC Patients

**Figure 1:**
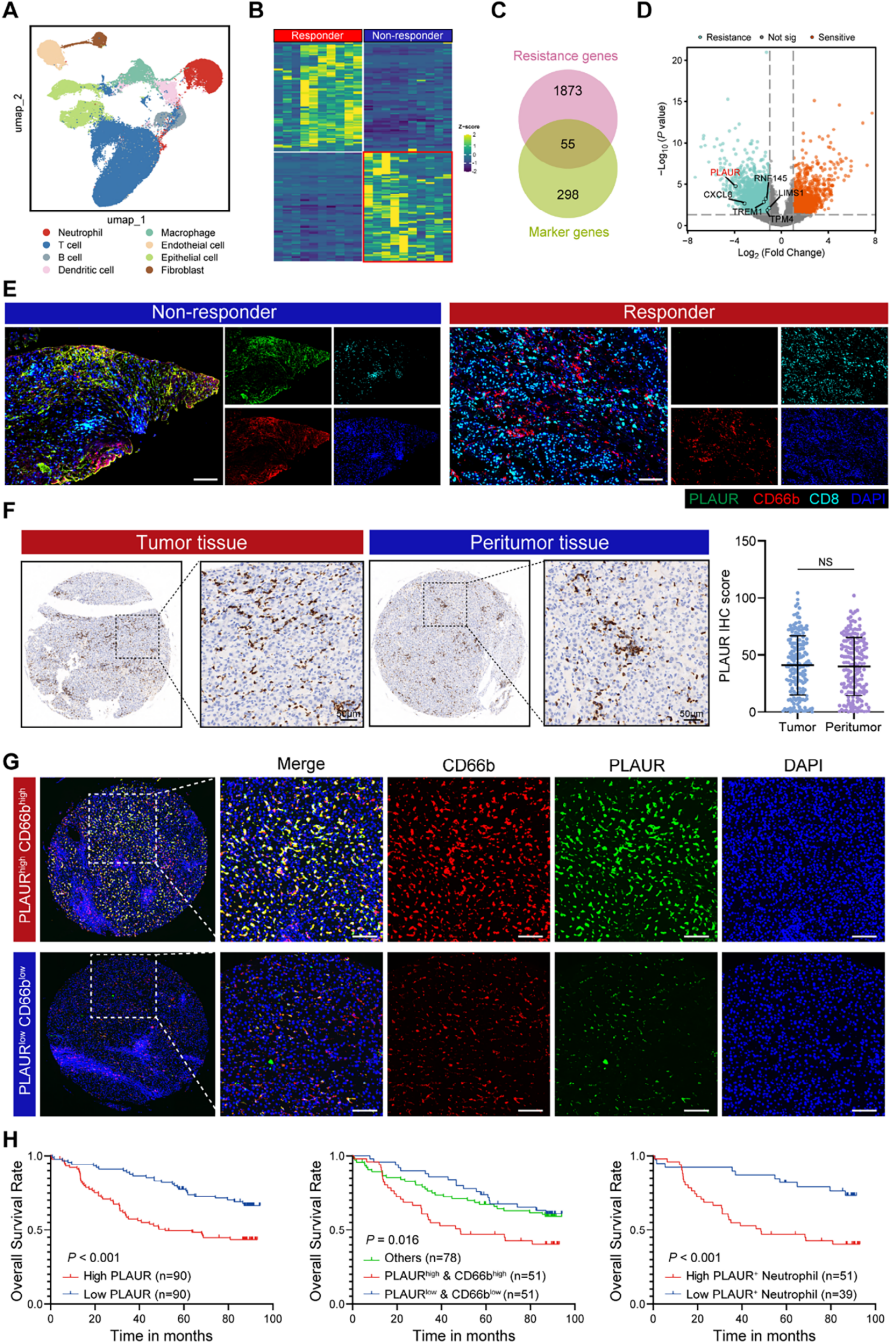
PLAUR^+^ neutrophils are correlated with the resistance to anti‐PD‐1 therapy and poor prognosis for HCC patients. A) UMAP plot for different clusters of single cells from PRJCA020880 and GSE202642 cohort. B) Heatmap of differentially expressed genes between anti‐PD‐1 therapy responders and non‐responders. C) Venn diagram of the overlapping potential neutrophil‐specific genes associated with the anti‐PD‐1 therapy resistance. D) Volcano plot of differentially expressed genes between anti‐PD‐1 therapy responders and non‐responders. E) Immunofluorescence staining of PLAUR, CD66b and CD8 in responders and non‐responders. Scale bar, 100 µm. F) IHC staining and statistical analysis of PLAUR expression in HCC tissues and peritumor tissues (*n* = 180). Scale bar, 50 µm. G) Immunofluorescence staining of PLAUR and CD66b in our patient cohort 2 (*n* = 180). Representative images from PLAUR^high^CD66b^high^ hHCC and PLAUR^low^CD66b^low^ hHCC were shown. Scale bar: 100 µm. H) OS curves for patients with HCC with high and low expression of PLAUR, PLAUR/CD66b co‐expressions and PLAUR^+^ neutrophils infiltration based on immunofluorescence staining in our patient cohort 2 (*n* = 180). The data are presented as the means ± SDs. *P < 0.05, **P < 0.01, and ***P < 0.001, Student's *t* test.

To comprehensively characterize the landscapes of neutrophils in the tumor microenvironment of HCC, we combined two public scRNA‐seq datasets (PRJCA020880 and GSE202642) for bioinformatics analyses. After the uniform manifold approximation and projection (UMAP) dimensional reduction analysis, copy number variation (CNV) analysis and examination of canonical marker genes, we obtained eight cell populations, including malignant cells, endothelial cells, T cells, B cells, fibroblasts, epithelial cells, macrophages and neutrophils (**Figures** **1**A and S1A, Supporting Information). Clustering analysis revealed significantly different transcriptional profiles among these cell population (Figure S1B, Supporting Information). We extracted 353 characteristic marker genes of neutrophils for further analysis. We confirmed the neutrophil specificity of these genes through Gene Ontology (GO) and Kyoto Encyclopedia of Genes and Genomes (KEGG) pathway analyses, which demonstrated their predominant involvement in neutrophil migration, phagosome, and endocytosis (Figure S1C,D, Supporting Information). We identified 55 neutrophil‐specific genes associated with resistance to anti‐PD‐1 therapy and conducted the least absolute shrinkage and selection operator (LASSO) analyses in the TCGA‐LIHC cohort to determine which of these genes significantly impact both resistance to anti‐PD‐1 therapy and the prognosis of patients with HCC (Figure 1B,C and Figure S2A,B, Supporting Information). This process identified six candidate neutrophil‐specific genes, among which PLAUR was selected for further investigation due to its predominant expression in neutrophils and significant upregulated in resistant tumors (Figure 1D and Figure S2C–G, Supporting Information). Spatial transcriptome analysis revealed that PLAUR^+^ neutrophils highly infiltrated in HCC patients with resistance to immunotherapy (Figure S3, Supporting Information). Besides, we performed immunofluorescence staining of tumor tissues from 42 HCC patients receiving anti‐PD‐1 therapy (Cohort 1) to further investigate the association between PLAUR^+^ neutrophils and anti‐PD‐1 therapy resistance. Notably, PLAUR^+^ neutrophils were predominantly present in anti‐PD‐1 therapy non‐responders (Figure 1E and Table S1, Supporting Information).

Next, we investigated the impact of PLAUR^+^ neutrophils on the prognosis of HCC patients. Analysis of the TCGA‐LIHC cohort showed no significant difference in PLAUR expression between tumor and peritumor tissues. However, HCC patients with high PLAUR expression were associated with poor tumor differentiation and experienced worse OS than those with low PLAUR expression (Figure S4, Supporting Information). Consistently, analysis of our cohort comprising 180 HCC patients (Cohort 2) demonstrated no significant difference in PLAUR expression between tumor and peritumor tissues, and high expression of PLAUR was linked to poor OS (Figure 1F,H). We further stratified HCC patients from Cohort 2 based on CD66b and PLAUR, as determined by immunofluorescence staining (Figure 1G). Tumors with low expression of both PLAUR and CD66b represented the best OS in HCC patients. Conversely, those with high infiltration of PLAUR⁺ neutrophils experienced worse OS compared to patients with lower infiltration levels (Figure 1H). These results indicated that PLAUR⁺ neutrophils are associated with resistance to anti‐PD‐1 therapy, and their presence in tumor tissues indicates worse OS of HCC patients.

### 2.2 Intratumoral PLAUR^+^ Neutrophils Diminish the Efficacy of Anti‐PD‐1 Therapy and Inhibits Infiltration of CD8^+^ T Cells

**Figure 2:**
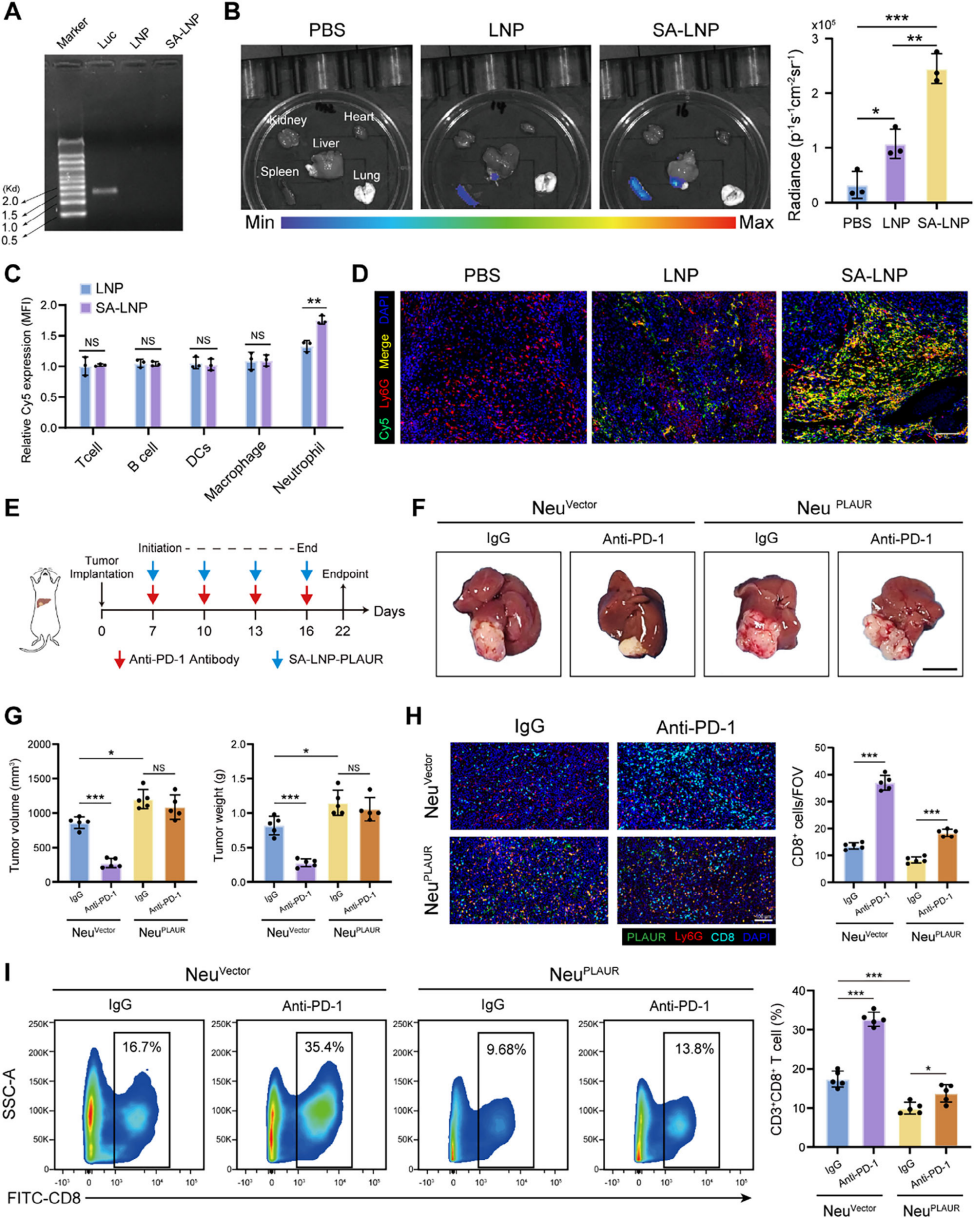
Intratumoral PLAUR^+^ neutrophils diminish the efficacy of anti‐PD‐1 therapy and inhibits infiltration of CD8^+^ T cells. A) The loading capacity of Luc‐mRNA by LNP and SA‐LNP was determined by agarose gel electrophoresis. B) Translation and quantification of Luc‐mRNA delivered by SA‐LNPs in major organs of tumor‐bearing mice after intravenous injection (*n* = 3 per group). C) Flow cytometry analysis of the expression of Cy5 in T cells, B cells, macrophages, DCs and neutrophils (*n* = 3 per group). D) Immunofluorescence staining of neutrophils in the liver tumor 6 h after intravenous injection of Cy5‐labeled SA‐LNPs or LNPs. Scale bars: 100 µm. E) Schematic showing the treatment plan and establishment of orthotopic HCC models in mice. F) Representative images of the orthotopic tumors at the study endpoint (5 mice per group). Scale bar: 1 cm. G) The tumor volume and tumor weight of each group at the study endpoint (*n* = 5 per group). H) Immunofluorescence staining and statistical analysis of PLAUR, Ly6G and CD8 in the indicated groups (*n* = 5 per group). Scale bars: 100 µm. I) Flow cytometry analysis of CD3^+^CD8^+^ T cells in the indicated groups (*n* = 5 per group). The data are presented as the means ± SDs. *P < 0.05, **P < 0.01, ***P < 0.001 and NS, not significant, One‐way ANOVA with a post hoc LSD test (or Student's *t* test in part C).

We constructed the neutrophil‐specific mRNA‐LNPs vaccine platform, which was formulated by mixing an ethanol phase containing MC3, DSPC, choline, mPEG2000‐DSPE (or sialic acid‐PEG2000‐DSPE) and SDEA with an aqueous phase containing nucleoside‐modified mRNAs. Dynamic light scattering (DLS) analysis revealed that the addition of sialic acid (SA‐LNP) did not significantly alter the size or zeta potential of the mRNA‐LNPs vaccine platform (Figure S5A,B, Supporting Information). Agarose gel assays confirmed nearly complete luciferase mRNA entrapment within SA‐LNPs (**Figure** **2**A). Next, we investigated the mRNA translation efficiency of SA‐LNPs. Imaging and quantitative analysis confirmed luciferase protein expression primarily in the liver and SA‐LNPs showed better translation efficiency (Figure 2B). To examine the hepatic neutrophils selectivity of the SA‐LNPs vaccine after intravenous injection, we substituted 33.3% of the cholesterol content with Cy5‐labeled cholesterol. These particles were administered intravenously to tumor‐bearing C57BL/6 mice, followed by the collection of IVIS images and flow cytometry analysis 6 h later to determine the in vivo biodistribution profiles of the SA‐LNPs vaccine platform. The results demonstrated that the SA‐LNPs significantly accumulated in the liver tumor and displayed improved hepatic neutrophil selectivity compared with LNPs, which was further verified by immunofluorescence staining of tissues (Figure 2C,D and Figure S5C,D, Supporting Information).

To investigate the effect of PLAUR⁺ neutrophils on the efficacy of anti‐PD‐1 therapy, we employed SA‐LNPs vaccine platform to establish orthotopic HCC mouse models bearing hepatic tumors formed from PLAUR‐overexpressing or control neutrophils and treated the mice with an anti‐PD‐1 mAb or IgG2a (Figure 2E). At the study endpoint, mice in the PLAUR‐overexpressing neutrophils group exhibited significantly increased tumor size and resistance to the anti‐PD‐1 mAb (Figure 2F,G). Immunofluorescence staining and flow cytometry analyses further revealed a marked reduction in CD8⁺ T cell infiltration in the PLAUR‐overexpressing neutrophils group (Figure 2H,I). Collectively, these results suggested that intratumoral PLAUR^+^ neutrophils diminish the efficacy of anti‐PD‐1 therapy and inhibits infiltration of CD8+ T cells.

### 2.3 Intratumoral PLAUR^+^ Neutrophils Correlate with Immune Exhaustion Landscape in HCC

**Figure 3:**
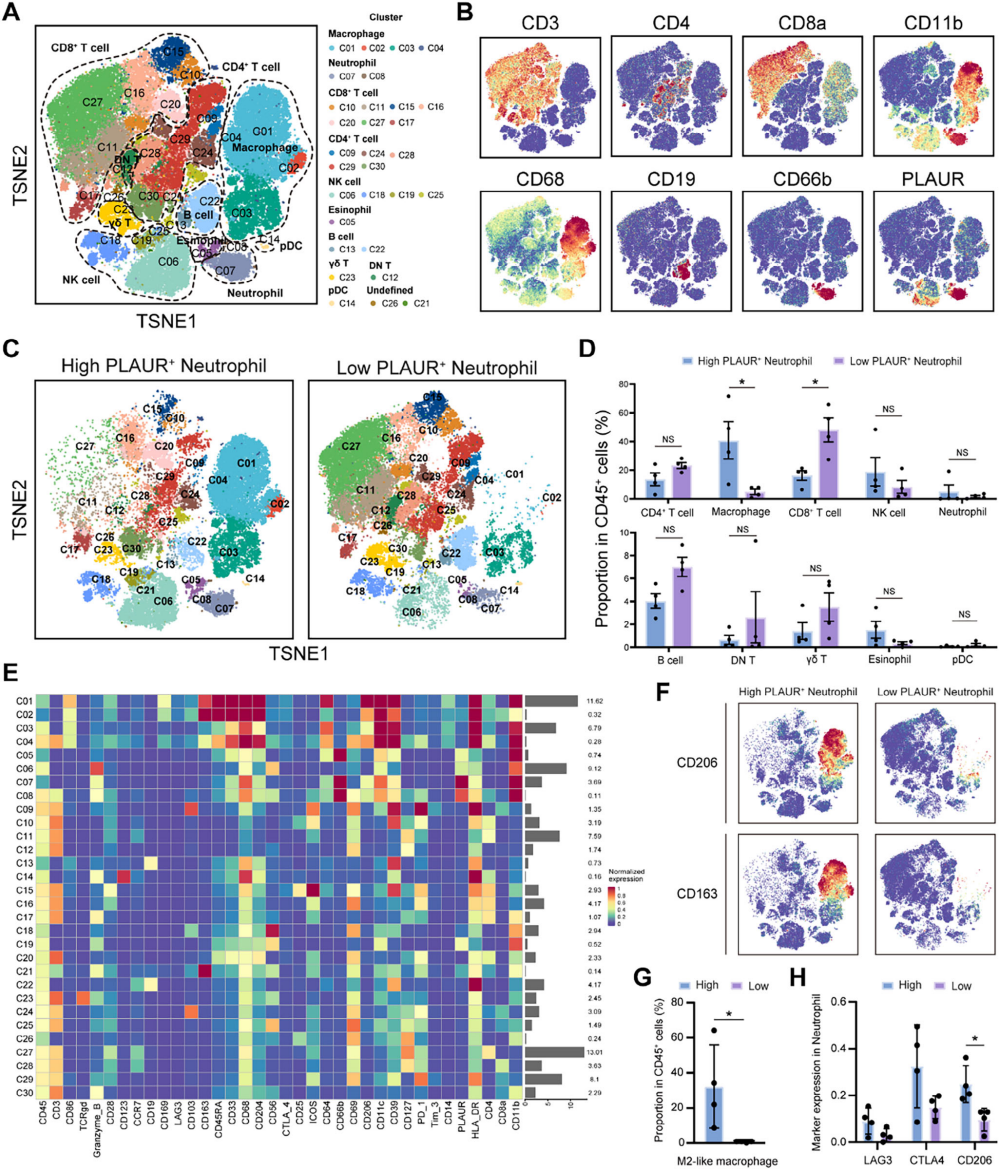
Intratumoral PLAUR^+^ neutrophils correlate with immune exhaustion landscape in HCC. A) t‐SNE plot of CD45^+^ cells in the tumor from eight patients with HCC based on CyTOF results. Cell cluster type was annotated according to the known marker expression. B) t‐SNE plot of CD45^+^ cells colored by the levels of CD3, CD4, CD8a, CD11b, CD19, CD66b, CD68, and PLAUR. C) t‐SNE plot of CD45^+^ cells in the tumors from PLAUR^+^ neutrophil low and high infiltration groups. D) Proportion of the indicated immune cell types in CD45^+^ cells in the two groups (*n* = 4 per group). E) Heatmap of 36 markers expressed in each cell cluster. F) The expression of CD206 and CD163, among CD45^+^ cells in the two groups. G) Proportion of M2‐like macrophage in CD45^+^ cells in the two groups (*n* = 4 per group). H) The expression level of LAG3, CTLA‐4 and CD206 in neutrophils of two groups (*n* = 4 per group). The data are presented as the means ± SDs. *P < 0.05, **P < 0.01, and ***P < 0.001, Student's *t* test.

To elucidate the mechanism underlying the immunotherapy resistance and poor prognosis in HCC patients with high infiltration of PLAUR^+^ neutrophils, we profiled immune cell infiltration and functional status between HCC tumors with high and low infiltration of PLAUR^+^ neutrophil using CyTOF analyses. Clustering analysis identified 27 distinct cell clusters across eight HCC tumors, which were annotated manually into 10 cell types including macrophages, neutrophils, CD8^+^ T cells, CD4^+^ T cell, etc., based on the known markers (**Figure** **3**A,B). We observed a significant increased infiltration of macrophages and reduced infiltration of CD8^+^ T cells in HCC tumors with high levels of intratumoral PLAUR^+^ neutrophils (Figure 2C,D). Further analysis of the marker expression showed that macrophages exhibited immunosuppressive characteristics in tumors with high levels of intratumoral PLAUR^+^ neutrophils (Figure 2E–G). In addition, neutrophils displayed elevated levels of LAG3, CTLA4 and CD206 expression in tumors with high level of intratumoral PLAUR^+^ neutrophils (Figure 2H). We also examined the associations of intratumoral PLAUR expression with the immune landscape in TCGA‐LIHC cohort. ssGSEA‐based cluster analyses revealed that tumors with high PLAUR expression demonstrated a distinct immunosuppressive phenotype compared to those with low PLAUR expression (Figure S6A, Supporting Information). Consistent with these findings, the CIBERSORT analysis of the TCGA‐LIHC cohort showed a positive correlation between intratumoral PLAUR levels and the infiltration of M2 macrophages (Figure S6B, Supporting Information). Taken together, these results indicated that PLAUR^+^ neutrophils exhibit an immunosuppressive state and may regulate tumor infiltration of macrophages and CD8^+^ T cell, thereby contributing to an immune exhaustion contexture in the HCC microenvironment.

### 2.4 PLAUR^+^ Neutrophils Exhibit an Immunosuppressive Phenotype and Affect Macrophages and CD8^+^ T Cells to Reshape the Immune Microenvironment

**Figure 4:**
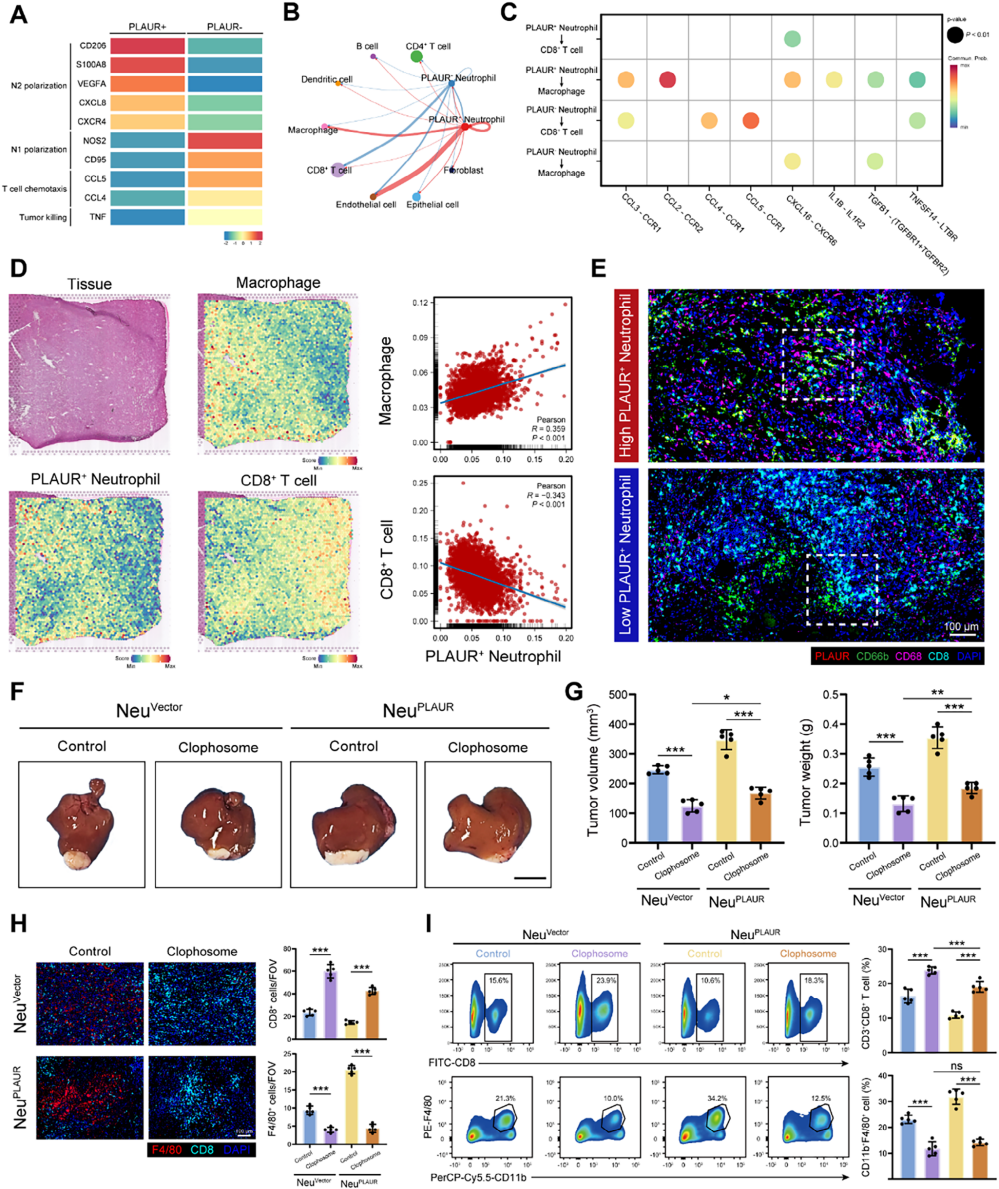
PLAUR^+^ neutrophils exhibit an immunosuppressive phenotype and affect macrophages and CD8^+^ T cells to reshape the immune microenvironment. A) Heatmap of the indicated genes in PLAUR^+^ neutrophils and PLAUR^−^ neutrophils. B) Cell–cell communications between PLAUR^+^/PLAUR^−^ neutrophils and other cell types. C) Dot plot showing ligand–receptor pairs between PLAUR^+^/PLAUR^−^ neutrophils and macrophages and CD8^+^ T cells. D) Distribution and Pearson correlation analysis of PLAUR^+^ neutrophils, macrophages and CD8^+^ T cells in HCC samples based on ST analyses. E) Immunofluorescence staining of PLAUR, CD66b, CD8 and CD68 in HCC patients. Scale bar, left: 100 µm. F) Representative images of the orthotopic tumors in the indicated groups (5 mice per group). Scale bar: 1 cm. G) The tumor volume and tumor weight of each group at the study endpoint (*n* = 5 per group). H) Immunofluorescence staining and statistical analysis of F4/80 and CD8 in the indicated groups (*n* = 5 per group). Scale bars: 100 µm. I) Flow cytometry analysis of CD3^+^CD8^+^ T cells and CD11b^+^F4/80^+^ macrophages in the indicated groups (*n* = 5 per group). The data are presented as the means ± SDs. *P < 0.05, **P < 0.01, ***P < 0.001, One‐way ANOVA with a post hoc LSD test.

We employed scRNA‐seq datasets to analyze the differences between PLAUR^+^ and PLAUR^−^ neutrophils. GO analysis indicated that PLAUR^+^ neutrophils are mainly involved in immune response‐regulating signaling pathway, cytokine‐mediated signaling pathway and immune receptor activity (Figure S7A, Supporting Information). Of note, KEGG analysis revealed that the nuclear factor kappa B (NF‐κB)/p65 signaling pathway, which is known to drive N2 polarization, was significantly activated in PLAUR^+^ neutrophils^[^ ^17^, ^18^, ^19^ ^]^ (Figure S7B, Supporting Information). Moreover, PLAUR^+^ neutrophils exhibited elevated expression of genes associated with N2 polarization, such as CD206, VEGFA, S100A8, and CXCL8, while the expression of genes related to T cell chemotaxis and tumor killing was markedly reduced (**Figures** **4**A and S8, Supporting Information). Next, we used CellChat to further investigate the biological signaling pathway involved in their interactions with macrophages and CD8⁺ T cells (Figure S9, Supporting Information). The network plot displayed that PLAUR^+^ neutrophils communicated preponderantly with macrophages and endothelial cells (Figure 4B). Furthermore, analysis of ligand–receptor interactions revealed that PLAUR⁺ neutrophils engaged in more extensive signaling with macrophages, but exhibited attenuated interactions with CD8⁺ T cells, compared to PLAUR⁻ neutrophils (Figure 4C). By integrating the ST dataset^[^ ^20^ ^]^ with the scRNA‐seq datasets, we found that regions with higher infiltration scores of PLAUR⁺ neutrophils corresponded with increased macrophage infiltration scores and decreased CD8^+^ T cell infiltration scores (Figure 4D). This spatial pattern was further validated by multiplex immunofluorescence staining of human HCC tissues, which demonstrated high infiltration of macrophages and less infiltration of CD8⁺ T cells surrounding PLAUR⁺ neutrophils (Figure 4E).

Given the impact of PLAUR^+^ neutrophils on CD8^+^ T cells and macrophages, we further explored whether the tumor progression induced by PLAUR^+^ neutrophils occurred in a CD8^+^ T cell‐dependent or macrophage‐dependent manner. We treated the mice with anti‐CD8 neutralizing antibodies to deplete CD8^+^ T cells. The results demonstrated that anti‐CD8 neutralizing antibody significantly abrogated the antitumor effect of PLAUR‐knockdown neutrophils (Figure S10A,B, Supporting Information). Immunofluorescence staining and flow cytometry analyses showed that CD8⁺ T cell depletion did not alter macrophage infiltration (Figure S10C,D, Supporting Information). To assess the role of macrophages, we next used Clophosome to deplete macrophages in mice. Notably, macrophage depletion diminished the effects of PLAUR⁺ neutrophils on tumor growth and CD8⁺ T cell infiltration, suggesting that macrophages play a critical role in the regulatory effect of PLAUR⁺ neutrophils on CD8⁺ T cell (Figure 4F–I). The CellChat analysis further revealed significant ligand‒receptor pairs between PLAUR^+^ neutrophils and macrophages, with C‐C motif chemokine ligand 2 (CCL2)–C‐C motif chemokine receptor 2 (CCR2) emerging as the most likely exclusive pair responsible for the binding of PLAUR neutrophils to macrophages (Figure 4C). Since CCL2 has been reported to promote tumor infiltration and M2 polarization of TAMs in HCC,^[^ ^21^ ^]^ we next investigated whether it mediates the effect of PLAUR^+^ neutrophils on macrophages. We observed that the addition of a CCR2 antagonist (RS102895) significantly attenuated the pro‐tumor effects of PLAUR^+^ neutrophils and increased the accumulation of M1‐like macrophages and CD8^+^ T cells within the tumor, which indicated that PLAUR^+^ neutrophils promoted the infiltration and M2‐like polarization of macrophages via the CCL2‒CCR2 axis (Figure S11, Supporting Information). Additionally, as illustrated by the IHC staining of Cohort 2, tumors with high PLAUR expression presented increased CD68 expression levels and decreased CD8 expression levels (Figure S12A,B, Supporting Information). Prognostic analysis revealed that PLAUR^low^/CD8^high^ and PLAUR^low^/CD68^low^ patients experienced the longest OS (Figure S12C, Supporting Information). Generally, we concluded that PLAUR^+^ neutrophils exhibit an immunosuppressive phenotype and reshape the tumor immune microenvironment by modulating macrophages and CD8⁺ T cells, contributing to poor prognosis in HCC patients.

### 2.5 PLAUR Inhibitor Reverses the Immunosuppressive Phenotype of Neutrophils and Attenuates Tumor Progression

**Figure 5:**
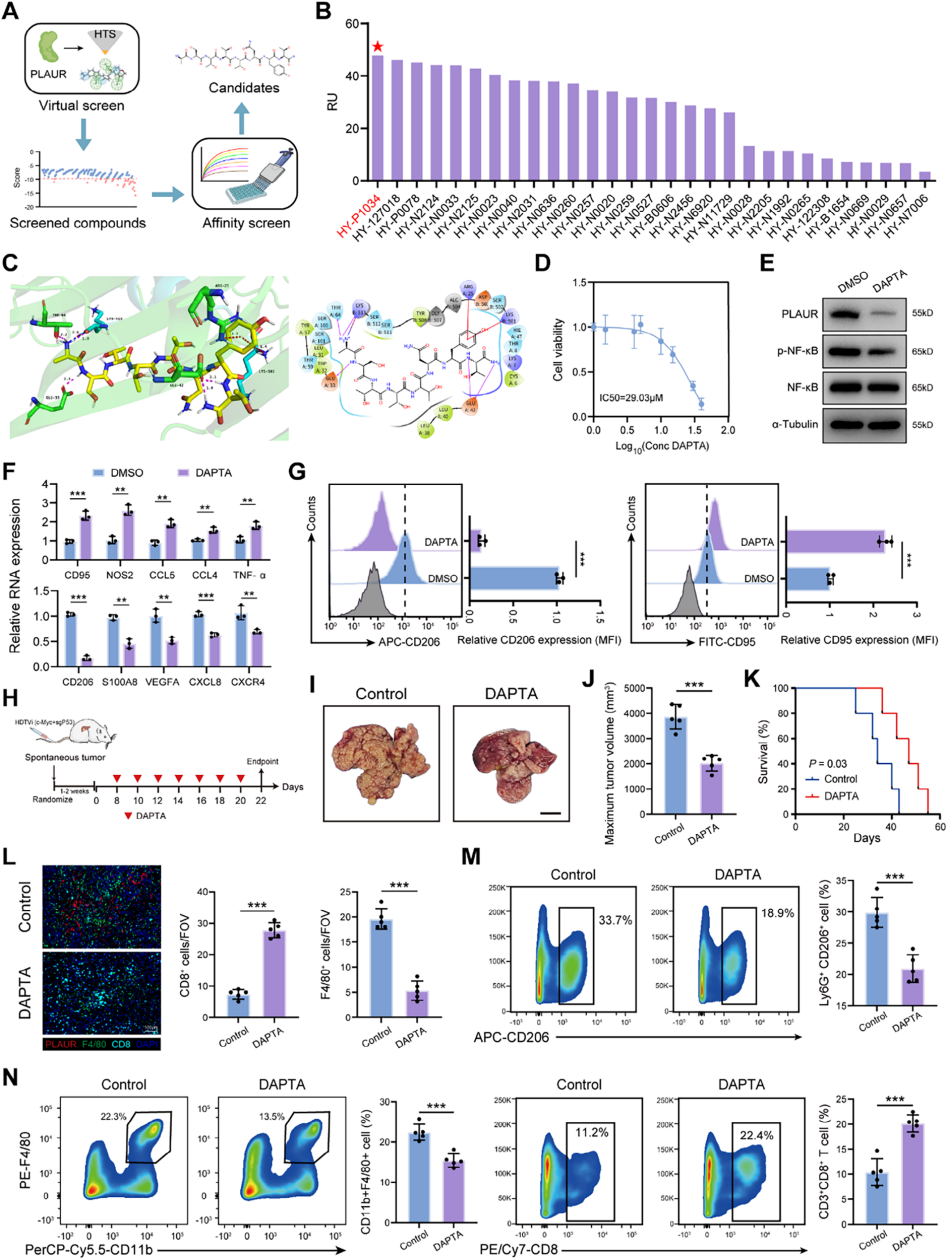
PLAUR inhibitor reverses the immunosuppressive phenotype of neutrophils and attenuates tumor progression. A) Flowchart depicting the screening strategy for small‐molecule compounds targeting PLAUR. B) The affinity of 40 candidate small‐molecule compounds targeting PLAUR detected by SPR analysis. C) Schematic diagram of the predicted docking structure of DAPTA and PLAUR. D) Cell viability of DAPTA detected in human neutrophils. E) Western blot analysis of PLAUR, phosphorylated and non‐phosphorylated NF‐κB expression in human neutrophils treated with DMSO or DAPTA (5 µm). F) quantitative PCR analysis of the indicated genes in DMSO and DAPTA treatment groups (*n* = 3 per group). G) Flow cytometry analysis of CD206 and CD95 expression in DMSO and DAPTA treatment groups (n = 3 per group). H) Schematic showing the treatment plan and establishment of spontaneous HCC models in mice. I) Representative images of the spontaneous tumors at the study endpoint (5 mice per group). Scale bar: 1 cm. J) The maximum tumor volume of each group at the study endpoint (*n* = 5 per group). K) Kaplan‐Meier survival curves for mice (*n* = 5 per group). L) Immunofluorescence staining and statistical analysis of PLAUR, F4/80 and CD8 in the indicated groups (*n* = 5 per group). Scale bars: 100 µm. M,N) Flow cytometry analysis of Ly6G^+^CD206^+^ neutrophils, CD11b^+^F4/80^+^ macrophages and CD3^+^CD8^+^ T cells in the indicated groups (*n* = 5 per group). The data are presented as the means ± SDs. *P < 0.05, **P < 0.01, and ***P < 0.001, Student's *t* test.

Currently, there are no commercially available PLAUR inhibitors. Therefore, we conducted a structure‐based high‐throughput virtual screening (HTVS) of 25361 compounds in search of potential PLAUR inhibitors (**Figure** **5**A). Based on the predicted binding affinity, forty top‐ranking compounds were selected for surface plasmon resonance (SPR) analysis. The results showed that D‐Ala‐peptide T‐amide (DAPTA; MedChemExpress No. HY‐P1034) exhibited the highest binding affinity to the PLAUR protein, forming hydrogen bonds at Glu33, Glu44, Thr64, and Lys513 (Figure 5B,C). The half‐maximal inhibitory concentration (IC50) in neutrophils was 29.03 µm (Figure 5D). Western blot demonstrated that DAPTA significantly reduced PLAUR protein levels and suppressed the activation of the NF‐κB/p65 signaling pathway in neutrophils (Figure 5E). In addition, DAPTA treatment remarkedly inhibited the expression of immunosuppressive genes in neutrophils, indicating that DAPTA reverses the immunosuppressive phenotype of neutrophils (Figure 5F,G). We performed a mass spectrometry analysis of neutrophils to further examine the molecular mechanism by which PLAUR activates the NF‐κB/p65 pathway and found that myosin heavy chain 9 (MYH9) is a potential PLAUR‐interacting protein that has been reported to activate the NF‐κB/p65 pathway^[^ ^22^ ^]^ (Figure S13A, Supporting Information). Coimmunoprecipitation analyses confirmed the interaction of PLAUR with MYH9 in neutrophils (Figure S13B, Supporting Information). Inhibition of PLAUR in neutrophils by DAPTA resulted in decreased MYH9 protein expression (Figure S13C, Supporting Information). Subsequently, we determined whether MYH9 participates in the PLAUR‐mediated activation of the NF‐κB/p65 pathway by treating neutrophils with MYH9 inhibitor Blebbistatin to disrupt the expression of MYH9. We found that MYH9 suppression significantly abrogated the effects of PLAUR on NF‐κB/p65 pathways, as well as on VEGFA and CXCL8 transcription in neutrophils (Figure S13D,E, Supporting Information). These results indicated that PLAUR induced activation of the NF‐κB/p65 pathway in a MYH9‐dependent manner.

Next, we constructed spontaneous HCC mouse models to investigate the effect of DAPTA on tumor progression (Figure 5H). We observed that DAPTA treatment significantly inhibited tumor growth, which was further validated in orthotopic HCC mouse models (Figure 5I–K and Figure S14A,B, Supporting Information). Immunofluorescence staining and flow cytometry analyses showed that DAPTA treatment remarkedly decreased the tumor infiltration of CD206^+^ neutrophils and macrophages, while concomitantly enhancing CD8⁺ T cell infiltration (Figure 5L–N). However, the DAPTA treatment did not suppress tumor growth in immunodeficient mouse models, suggesting that the antitumor effects of DAPTA are exerted in an immune‐dependent manner (Figure S14C,D, Supporting Information). Collectively, these results identify DAPTA as a promising PLAUR‐targeting compound that effectively reverses the immunosuppressive phenotype of neutrophils and exerts immune‐dependent antitumor effects.

### 2.6 PLAUR Inhibitor Potentiates the Efficacy of Anti‐PD‐1 Antibody in HCC Preclinical Models

**Figure 6:**
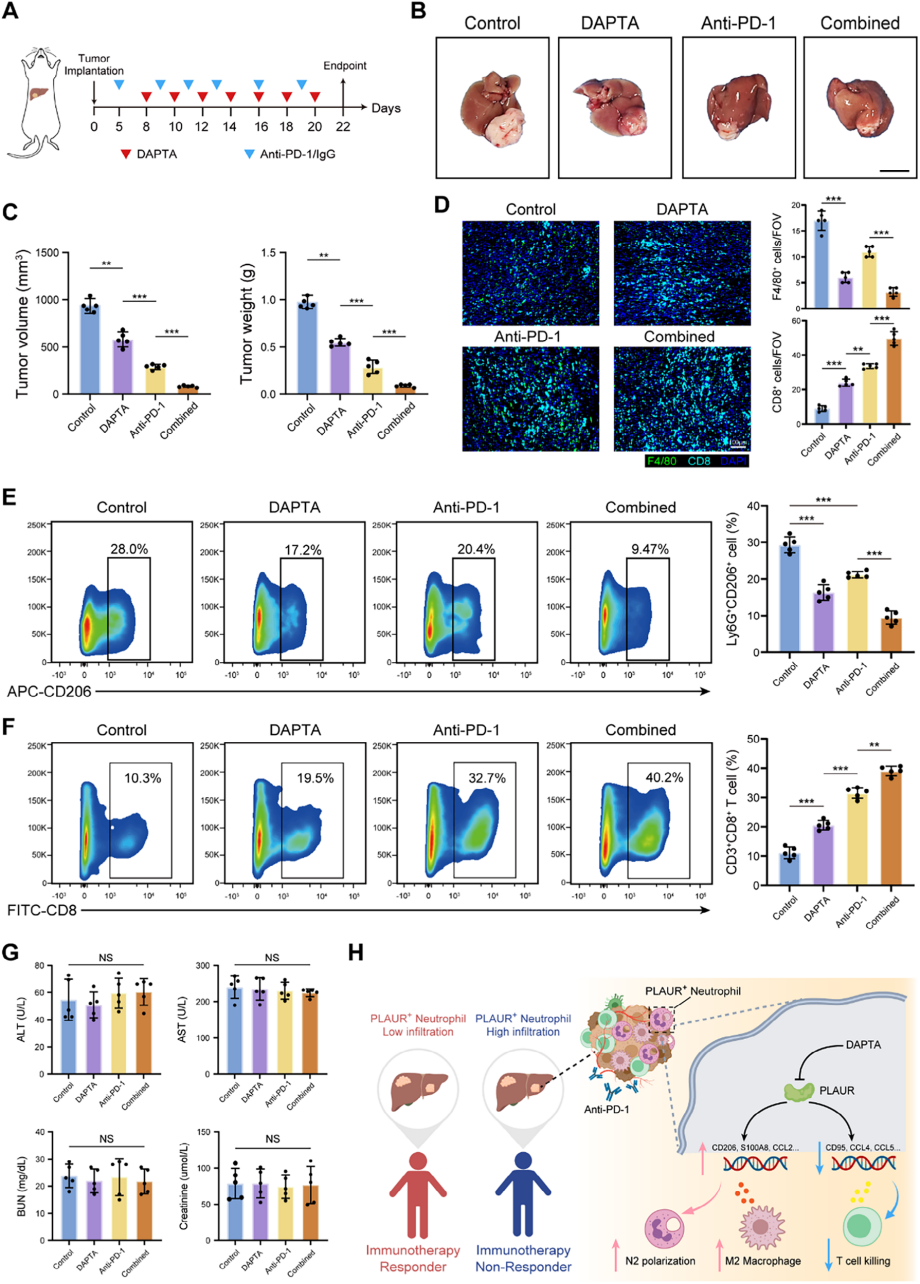
PLAUR inhibitor potentiates the efficacy of anti‐PD‐1 antibody in HCC preclinical models. A) Schematic showing the treatment plan and establishment of orthotopic HCC models in mice. B) Representative images of the orthotopic tumors at the study endpoint (5 mice per group). Scale bar: 1 cm. C) The tumor volume and tumor weight of each group at the study endpoint (*n* = 5 per group). D) Immunofluorescence staining and statistical analysis of F4/80 and CD8 in the indicated groups (*n* = 5 per group). Scale bars: 100 µm. E,F) Flow cytometry analysis of Ly6G^+^CD206^+^ neutrophils and CD3^+^CD8^+^ T cells in the indicated groups (*n* = 5 per group). G) Liver and kidney functions of mice with HCC tumors in each group at the study endpoint (*n* = 5 per group). H) Schematic diagram of the regulatory role of PLAUR in neutrophils and its impact on the immune landscape of the HCC microenvironment. The data are presented as the means ± SDs. *P < 0.05, **P < 0.01, ***P < 0.001 and NS, not significant, One‐way ANOVA with a post hoc LSD test.

The above findings drove us to further investigate whether PLAUR inhibition could improve the efficacy of immunotherapy in HCC models, we established orthotopic HCC mouse models and treated them with IgG, anti‐PD‐1 antibody, DAPTA, and anti‐PD‐1 antibody combined with DAPTA, respectively (**Figure** **6**A). At the study endpoint, we found that anti‐PD‐1 antibody combined with DAPTA treatment inhibited tumor growth more than the control treatment or single treatment alone (Figure 6B,C). Immunofluorescence staining and flow cytometry analyses further revealed that the combination therapy induced a significant immune response with increased infiltration of CD8^+^ T cells and decreased CD206^+^ neutrophils and macrophages into tumors (Figure 6D–F). No significant hepatic or renal toxicities were observed at the study endpoints for either monotherapy or the combination therapy (Figure 6G). Collectively, these findings indicated that the inhibition of PLAUR by DAPTA may enhance therapeutic efficacy of anti‐PD‐1 therapy in HCC.

## 3 Discussion

Although anti‐PD‐1 therapy has demonstrated success in various solid tumors, its therapeutic benefits in HCC patients remain restricted to specific subgroups. Neutrophils constitute a predominant immune cell population within the HCC microenvironment, participating in intricate and dynamic interactions with other cellular components that critically influence the tumor immune landscape and regulate therapeutic responses to immunotherapy.^[^ ^23^, ^24^ ^]^ This biological prominence has positioned neutrophil‐targeting strategies as a promising approach to enhance ICIs therapy in HCC. The present study elucidated the pivotal role of PLAUR^+^ neutrophils in mediating resistance to anti‐PD‐1 therapy and shaping an immunosuppressive TME in HCC. By integrating multi‐omics analyses, preclinical models, and clinical cohorts, we demonstrate that PLAUR^+^ neutrophils contributed to immune evasion and poor prognosis in HCC. These findings expand our understanding of neutrophil heterogeneity in HCC and highlight a novel therapeutic target to overcome immunotherapy resistance.

Previous studies have described the association of PLAUR with the clinical prognosis of patients with various cancers, but its exact function in the TME remains elusive.^[^ ^12^, ^13^, ^14^, ^15^, ^25^ ^]^ The present study identified PLAUR as a neutrophil‐specific marker associated with resistance to anti‐PD‐1 therapy and poor prognosis in HCC patients. Transcriptomic and spatial analyses revealed that PLAUR^+^ neutrophils were enriched in tumors of anti‐PD‐1 non‐responders. By establishing orthotopic HCC mouse models bearing hepatic tumors formed from PLAUR‐overexpressing or control neutrophils, we confirmed that intratumoral PLAUR^+^ neutrophils diminished the efficacy of anti‐PD‐1 therapy and decreased the infiltration of CD8^+^ T cells. The CyTOF analysis also observed a significant increased M2‐like macrophage infiltration and reduced CD8^+^ T cells infiltration in HCC tumors with high infiltration of PLAUR^+^ neutrophils. This aligns with emerging evidences that neutrophils can adopt immunosuppressive phenotypes, often termed N2 polarization, which hinder antitumor immunity.^[^ ^26^, ^27^ ^]^ The scRNA‐seq data further delineated the molecular landscape of PLAUR^+^ neutrophils, showing their engagement in NF‐κB/p65 signaling—a pathway known to drive immunosuppressive cytokine production and N2 polarization. The elevated expression of CD206, VEGFA, and CXCL8 in PLAUR^+^ neutrophils suggested a dual role in promoting angiogenesis and recruiting immunosuppressive macrophages.

Mechanistically, PLAUR^+^ neutrophils appeared to orchestrate immune suppression through crosstalk with macrophages. The CellChat analysis and depletion experiments revealed that macrophages were critical role in the PLAUR^+^ neutrophil‐mediated inhibition of CD8^+^ T cells. This is consistent with prior studies showing that TAMs, particularly M2‐like subsets, suppress cytotoxic T lymphocyte activity and anti‐inflammatory cytokines.^[^ ^7^, ^28^ ^]^ Our study suggested that PLAUR^+^ neutrophils may recruit M2‐like macrophages by CCL2‐CCR2 axis to amplify the immunosuppressive TME. The dependency on macrophages for PLAUR^+^ neutrophil‐mediated immunosuppressive effects was further validated by the abrogation of tumor growth inhibition upon macrophage depletion and CCL2‐CCR2 axis blockade. Furthermore, the correlation among PLAUR, CD68, and CD8 expression in human HCC tissues suggests a functionally significant interaction that contributed to the poor survival duration of HCC patients. These findings position PLAUR^+^ neutrophils as important regulators of the HCC immune landscape, driving immune escape.

The translational implications of these findings are underscored by the development of DAPTA, a novel PLAUR inhibitor identified through structure‐based virtual screening. DAPTA effectively reversed the immunosuppressive phenotype of neutrophils by suppressing NF‐κB activation and downregulating genes associated with N2 polarization. In preclinical models, DAPTA treatment reduced tumor growth and increased CD8^+^ T cell infiltration. Notably, combining DAPTA with anti‐PD‐1 therapy displayed synergistic antitumor activity, overcoming the limitations of single‐agent immunotherapy. The lack of hepatotoxicity or nephrotoxicity in treated mice further supports the clinical feasibility of this approach.

Targeting PLAUR⁺ neutrophils in HCC represents a distinct yet complementary approach compared to other myeloid‐targeting strategies such as CSF1R or CXCR2 inhibitors. While CSF1R inhibitors primarily aim to deplete or reprogram immunosuppressive TAMs by blocking macrophage survival and differentiation, and CXCR2 inhibitors focus on limiting neutrophil recruitment by antagonizing chemokine‐driven trafficking,^[^ ^29^, ^30^, ^31^ ^]^ PLAUR inhibition selectively disrupts the immunosuppressive functions of N2‐like neutrophils without globally depleting neutrophils, potentially preserving beneficial neutrophil activities. Mechanistically, PLAUR blockade reverses NF‐κB‐driven immunosuppressive pathways, thereby reprogramming neutrophils rather than eliminating them. Moreover, PLAUR inhibition demonstrated synergistic effects with anti‐PD‐1 therapy in preclinical models, highlighting its potential as a combination strategy.

One limitation of our study is that the precise mechanisms by which PLAUR modulated NF‐κB activation in neutrophils and its interaction with macrophages warrant further investigation. Additionally, the association between PLAUR^+^ neutrophils and the immunotherapy resistance were evaluated in a small cohort of HCC patients receiving immunotherapy; thus, we aim to conduct a large‐scale, multicenter patient cohort study to obtain a more comprehensive understanding of this association and the implications for treatment in the future.

In conclusion, this study defines PLAUR^+^ neutrophils as critical mediators of immunotherapy resistance in HCC by forming an immunosuppressive TME characterized by CD8^+^ T cell exclusion and macrophage‐dependent immune suppression. The development of DAPTA, a novel PLAUR inhibitor, represents a promising therapeutic strategy to improve the efficacy of anti‐PD‐1 therapy.

## 4 Experimental Section

### Patients and Specimens

Cohort 1 containing 42 tumor tissues obtained from patients who received anti‐PD‐1 therapy at the Affiliated Cancer Hospital of Zhengzhou University for immunofluorescence staining. The characteristics of 42 HCC patients are summarized in Table S1 (Supporting Information). Cohort 2 containing 180 tumor and paired peri‐tumor tissues obtained from HCC patients who underwent curative resection from July 2002 to December 2006 at the Affiliated Cancer Hospital of Zhengzhou University and was used for immunofluorescence staining, immunohistochemistry (IHC) and survival analyses. HCC diagnosis was based on histology. Additionally, transcriptome data of tumor tissues from 20 HCC patients with different responses to anti‐PD‐1 therapy were obtained from our previous studies.^[^ ^32^, ^33^ ^]^ Tumor response was assessed according to the modified Response Evaluation Criteria in Solid Tumors,^[^ ^34^ ^]^ and responders and non‐responders were defined as previously described.^[^ ^33^ ^]^ The study protocol was approved by the research ethics committee of the Affiliated Cancer Hospital of Zhengzhou University and performed in accordance with the principles set by the Declaration of Helsinki. All patients signed written informed consent for therapy and study inclusion.

### Animal Studies

For the subcutaneous tumor model, Hepa1‐6 cells (5 × 10^6^ cells in 100 µL PBS) were subcutaneously injected into the right inguinal fold regions of C57BL/6 mice or NOD/SCID mice. For the orthotopic tumor models, subcutaneous Hepa1‐6 tumors from C57BL/6 mice were resected and cut into 1 mm^3^ pieces, which were subsequently implanted into the liver parenchyma of C57BL/6 mice that were anesthetized via anesthesia. For the spontaneous tumor model, a total of 30 µg of pT3‐EF1α‐MYC, 30 µg of px330‐sg‐p53, and SB13 transposase plasmid (at a 4:1 plasmid‐to‐transposase ratio) were dissolved in 0.9% NaCl solution. Each mouse received 2 mL of the plasmid/NaCl mixture via hydrodynamic tail vein injection (within 3–5 s).^[^ ^35^ ^]^ Mice were randomly assigned to groups according to the mean tumor volume. An ultrasound imaging platform and mouse MRI system were used to measure the tumors. For overexpression and knockdown of PLAUR in intratumoral neutrophil, sialic acid‐anchored RNA‐LNPs were injected intravenously twice a week after tumor implantation. For anti‐PD‐1 antibody intervention, mice were treated intraperitoneally with anti‐PD‐1 antibody (Bio X Cell, 200 µg per mouse) or IgG control following the schedule shown in Figures 2E and 6A. For the macrophages depletion in vivo, mice were treated intraperitoneally with Clophosome (FormuMax, 200 µL per mouse) every five days after tumor implantation. For cytokine receptor blockade in vivo, mice were injected intraperitoneally with CCR2 antagonist RS102895 (MedChemExpress, 100 µg per mouse) or vehicle control daily after tumor implantation. For the CD8^+^ T cells depletion in vivo, mice were treated intraperitoneally with anti‐CD8α antibody (Bio X Cell, 100 µg per mouse) every 3 d after tumor implantation. For PLAUR inhibitor treatment, mice were treated intraperitoneally with D‐Ala‐peptide T‐amide (DAPTA) (MedChemExpress, 100 µg per mouse) following the schedule shown in Figures 5H and 6A. At the endpoint of experiments, mice were sacrificed and tumors were dissected for further analyses.

All mice were maintained under specific pathogen‐free conditions and routinely monitored. All anesthesia or euthanasia methods were conducted in accordance with the Animal Research: Reporting of In Vivo Experiments (ARRIVE) guidelines and the study was approved by the Ethics Review Committee of the Animal Center, Zhengzhou University.

### Public Database Analyses

Single‐cell RNA sequencing (scRNA‐seq) data of patients with liver cancer cohort (PRJCA020880 and GSE202642) were obtained from the China National Center for Bioinformation and the Gene Expression Omnibus (GEO) website. Raw count matrices were merged and analyzed using the Seurat R package.^[^ ^36^ ^]^ Based on the quality control metrics suggested in the Scanpy tutorial,^[^ ^37^ ^]^ cells with less than 200 genes expressed were filtered out. Cells expressing more than 6000 genes, and more than 10% mitochondrial genes were also removed to ensure only the high quality of cells were used in the downstream analyses. Genes expressed in less than 3 cells were also filtered out of the analysis. Scaled and centered read counts were used as gene expression for further analysis. Subsequently, cell annotation was performed by examining highly expressed marker genes between clusters as well as literature‐derived and database‐derived cell markers (Figure S1A, Supporting Information). Marker genes for each cell cluster were determined by using the FindAllMarkers function of the Seurat pipeline. The differentially expressed genes between PLAUR^+^ and PLAUR^−^ fibroblasts were determined by using the FindMarkers function of the Seurat pipeline.

In addition, CellChat^[^ ^38^ ^]^ was used to examine the molecular interaction networks among various cell types. CellChat serves as a quantitative tool for inferring and analyzing intercellular communication networks derived from scRNA‐seq data. The findings from CellChat were utilized to uncover the incoming communication patterns of target cells and the outgoing communication patterns of secreting cells. Ligand‐receptor pairs identified by CellChat with a *p*‐value below 0.05 were deemed significant interacting molecules across different subsets.

For spatial transcriptomics (ST) analysis, R package Seurat was utilized for handling and illustrating ST data. ST data of patients with liver cancer cohort (GSE238264 and HRA000437) were obtained from the Gene Expression Omnibus (GEO) website and Genome Sequence Archive (GSA). To ascertain the proportions of cell subsets within each captured spot, SPOTlight deconvolution analysis was conducted following the guidelines provided at https://github.com/MarcElosua/SPOTlight. Subsequently, the marker gene matrix was identified for each cell subset from the scRNA‐seq dataset using Seurat's FindAllMarkers function with the criteria logfc.threshold = 1 and min.pct = 0.8. This marker gene matrix along with the ST data was inputted into the spotlight_deconvolution function utilizing its default settings. The outcomes of the deconvolution were integrated into the histological images using the spatial_scatterpie function, where each spot was labelled according to its subset. Following this, SpatialFeaturePlot was applied to depict the infiltration scores for each subset. Pearson correlation analysis was used to calculate the infiltration relationship of different subsets with each spot. All statistical analyses were conducted using R software (V.4.3.2).

### Statistical Analysis

All analyses were performed with GraphPad Prism 9 and R software (V.4.3.2). Numerical variables were shown as mean±SD and categorical variables as *n* (%). Differences between groups were tested using Student's *t* test or One‐way ANOVA with a post hoc LSD test, and constituent ratios were compared using the Pearson's chi‐square test or Fisher's exact test. The Kaplan‐Meier method with the log‐rank test was used for survival analysis. A two‐ tailed *P* value less than 0.05 was considered statistically significant.

More details of materials and methods are provided in the Supporting Information.

## Conflict of Interest

The authors declare no conflict of interest.

## Author Contributions

S.L., Y.Z., G.L., and B.Z. contributed equally to this study. S.‐Q.L., Y.‐Z.Z., G.‐X.L. and B.‐W.Z. performed experiments, analyzed data, and wrote the manuscript. S.‐Q.L., Y.‐Z.Z., and F.W. contributed to the animal experiments. S.‐Q.L., G.‐X.L., and J.‐X.Z. contributed to the acquisition and interpretation of data. S.‐Q.L., Y.‐Z.Z., and X.‐B.C. provided patient samples and clinical data. S.‐Q.L., Y.‐Z.Z., B.Q., and Y.‐X.G. provided scientific input and revised the manuscript. W.‐X.X., Y.J., and F.‐Z.W. supervised and conceived the entire project. All authors take full responsibility for the integrity of the data and the accuracy of the analysis.

## Ethics Approval

The study was reviewed and approved by the research ethics committee of the Affiliated Cancer Hospital of Zhengzhou University and the Ethics of the Animal Center, Zhengzhou University. The participants gave informed consent to participate in the study before taking part.

## Supporting information



Supporting Information

## Data Availability

All data relevant to the study are included in the article or uploaded as Supporting Information. The data are available from The Cancer Genome Atlas (http://cancergenome.nih.gov/) and GSE202642 in the GEO (Gene Expression Omnibus) website (https://www.ncbi.nlm.nih.gov/geo/) and PRJCA020880 in the China National Center for Bioinformation. All data relevant to the study are included in the article or uploaded as Supporting Information.
