# Supplementary material for: PLAUR+ Neutrophils Drive Anti‐PD‐1 Therapy Resistance in Patients with Hepatocellular Carcinoma by Shaping an Immunosuppressive Microenvironment
Source: Adv Sci (Weinh). 2025 Jul 17;12(38):e07167. doi: 10.1002/advs.202507167 (PMC12520528; doi:10.1002/advs.202507167)
Supplement: Supplementary file 1 — Supporting Information [file ADVS-12-e07167-s001.docx]

**Supplemental Data**

**PLAUR^+^ Neutrophils Drive Anti-PD-1 Therapy Resistance in Patients with Hepatocellular Carcinoma by Shaping an Immunosuppressive Microenvironment**

**The Supplemental Data consist of:**

Supplementary materials and methods

Supplementary figures S1-S14

Supplementary tables S1-S4

**Supplementary materials and methods**

**Materials**

DLin-MC3-DMA, cholesterol and DSPC were purchased from AVT (Shanghai) Pharmaceutical Tech Co., Ltd. Sialic acid-PEG_2000_-DSPE and mPEG_2000_-DSPE were supplied by Shanghai Ponsure Biotech, Inc. Phosphate buffer (PBS) and citrate buffer were purchased from Beijing Solarbio Science & Technology Co., Ltd. D-Luciferin was purchased from Biovision Biotechnology. N1-Me-Pseudo UTP, the T7 High Yield RNA Transcription Kit, Cap 1 Capping System and E. coli Poly(A) Polymerase were acquired from Novoprotein, shanghai, China. Cy5-modified N1-Me-Pseudo UTP was purchased from Jiangsu Synthgene Biotechnology Co., Ltd. CD87-siRNA was purchased from GenePharma, Shanghai, China.

**Preparation and characterization of sialic acid-anchored RNA-LNPs**

PLAUR mRNA and Cy5-modifid firefly luciferase mRNA were produced by in vitro translation (IVT), according to the kit instructions provided by the manufacturer. RNA-loaded LNPs were then prepared by mixing the lipid solutions in ethanol and RNA solutions in citrate buffer as previously described(1,2). Sialic acid-anchored RNA-LNPs were obtained with the similar procedure by replacing the mPEG_2000_-DSPE with sialic acid-PEG_2000_-DSPE (Ponsure Biotech). Particle size and zeta potential of RNA-loaded LNPs were measured using a Zetasizer Nano ZS90 (Malvern Instruments). The mRNA loading efficiency of LNPs and sialic acid-anchored LNPs (T-LNPs) was assessed using denaturing formaldehyde agarose gel electrophoresis.

**Selectivity of sialic acid-anchored RNA-LNPs by neutrophils in liver tumor**

The translation and biodistribution of Firefly luciferase mRNA-loaded sialic acid-anchored LNPs were performed with tumor-bearing female C57BL/6 mice. D-luciferin was administered intraperitoneally in a volume of 100 μL (33 mg/mL) per mouse 6 h after the intravenous injection of mRNA-LNPs. The mice were killed 5 min after the injection of D-luciferin, and the organs of heart, liver, spleen, lung, and kidney were harvested. The translation and biodistribution of Firefly luciferase mRNA in major organs of tumor-bearing mice was immediately recorded using the IVIS lumina II system (PerkinElmer, Waltham, MA).

To investigate the selectivity of sialic acid-anchored LNPs by neutrophils in liver tumor, the single-cell suspensions of liver tumor tissue were obtained and then stained with 7-AAD, anti-CD45, anti-CD11b and anti-Ly6G. The cellular uptake of Firefly luciferase mRNA-loaded sialic acid-anchored LNPs by neutrophils were analyzed by flow cytometry.

**Cytometry by time-of-flight (CyTOF)**

Fresh tumor tissues excised from ten patients with HCC were prepared into single cell suspensions and then incubated with 36 metal-conjugated antibodies. Subsequently, signal detection was performed in the Helios3 CyTOF system by PLTTech (Hangzhou, China). The CyTOF data was then normalized and analyzed on the Cytobank platform. Unsupervised clustering and t-SNE dimensionality reduction were performed based on the expression profiles of these markers using the ‘cytofkit’ package in R software to identify cell types. For data analysis, samples were stratified into high and low groups based on the median proportion of PLAUR^+^ neutrophil. Detailed antibody information was summarized in Supplementary Table S2.

**Immunofluorescence assay**

Slides were deparaffinized and rehydrated in xylene and graded ethanol. After being incubated with 0.3% hydrogen peroxide and retrieved antigen with citrate buffer, tissues were blocked with 5% BSA. Next, primary antibodies were used to incubate tissues overnight at 4℃. The primary antibodies were summarized in Supplementary Table S4. The next day, the corresponding secondary horseradish peroxidase-conjugated antibody was used to incubate the washed tissues and the slides were placed in citrate buffer to remove redundant antibodies. At last, the slides were incubated with DAPI solution at 37 ℃ for 15 mins without light. Slides were captured and scanned employing a slice scanner (Pannoramic MIDI: 3Dhistech, Hungary) and images were evaluated using HALO 2.0 Area Quantification algorithm (Indica Labs; Corrales, NM), at Nanjing Freethinking Biotechnology Co., Ltd. (Nanjing, China).

**Immunohistochemistry (IHC)**

Slides were deparaffinized and rehydrated, followed by antigen retrieval using EDTA buffer. After blocked with 5% BSA for 30 min, samples were sequentially incubated with primary antibodies and an HRP-conjugated secondary antibody. The protein expression levels were assessed by two experienced pathologists blinded to the clinical data using a semiquantitative method(3). Patients were classified into low and high expression groups based on the median expression. The antibodies used for IHC staining were present in the Supplementary Table S4.

**Flow cytometry**

Fresh tumor samples from mouse models were cut into small pieces and prepared into single cell suspensions using the Tumor Dissociation Kit (Miltenyi Biotec) as described above. Cultured cells were trypsinized and resuspended in the PBS buffer. After stained with the fixable viability dye (BioLegend) and permeabilized using Fixation and Permeabilization Solution (BD Biosciences), cells were incubated with fluorochrome-conjugated antibodies on ice for 30 min in the dark. Data acquisition was performed on a BD FACSAria Flow Cytometer, and further analyzed using FlowJo V10 software. The antibodies used were listed in the Supplementary Table S4.

**Western blot analysis**

Proteins were extracted from cultured cells using radio immunoprecipitation assay (RIPA) buffer and quantified using the Bicinchoninic Acid (BCA) assay kit. After thermal denaturation, proteins were separated by sodium dodecyl sulfate polyacrylamide gel electrophoresis (SDS-PAGE) and transferred onto Polyvinylidene Fluoride (PVDF) membranes according to the standard procedure. Later, bands were blocked with 5% skim milk and incubated overnight with the corresponding primary antibody. Finally, the blots were exposed with chemiluminescence reagents after incubating with the HRP-conjugated secondary antibody. The antibodies used were listed in Supplementary Table S4.

**Quantitative Real-Time Polymerase Chain Reaction (qRT-PCR)**

Total RNA was extracted utilizing RNAiso Plus (Takara) following the manufacturer’s instructions, and then reverse transcribed with the RT reagent Kit with gDNA Eraser (Takara). Quantitative real-time PCR were performed in triplicate using a LightCycler 480 (Roche Diagnostics). Results were normalized according to the mRNA expression of internal control GAPDH, and the comparative ΔΔCt method was used to calculate the relative expression of genes. The primers used were listed in Supplementary Table S3.

**Cell lines and cell culture**

The murine HCC cell line Hepa1-6 were purchased from the Stem Cell Bank, Chinese Academy of Science (Shanghai China). Cells were cultured in Dulbecco’s modified Eagle’s medium (DMEM, Gibco) supplemented with 10% foetal bovine serum (FBS, Gibco) and 100 U/L penicillin/streptomycin (Gibco). Human peripheral blood neutrophils isolation was performed according to previous description(4). Peripheral blood was collected from healthy volunteers into EDTA-coated tubes. First, neutrophils were isolated using MojoSort™ Whole Blood Human Neutrophil Isolation Kit (Biolegend). The obtained neutrophils were resuspended in RPMI 1640 medium (Gibco) supplemented with 10% fetal bovine serum, 1% penicillin, and streptomycin, seeded in 6-well plates. Cells were maintained at 37 ℃ in a humidified incubator with 5% CO_2_.

**Structure-based virtual screening**

We obtained the PLAUR protein structure (PDB ID: 1YWH) from the Protein Data Bank. To identify promising compounds, virtual screening was conducted using a commercial chemical library, each containing 25361 compounds. The screening process employed grid‐based ligand docking facilitated by the GLIDE software (Schrödinger Maestro 11.4), meticulously targeting the expected locations. Subsequently, the top forty compounds with the highest scores from MedChemExpress for experimental investigation were procured.

**Surface plasmon resonance (SPR) assays**

The PLAUR protein was separately diluted in 10 mM sodium acetate (pH 4.5) to a concentration of 200 μg/ml. Then, the protein was immobilized on a CM5 chip using the amine coupling kit to a final concentration of ∼10,000 response units (RU)/flow cell. SPR measurements were performed at 25 °C using a Biacore 8K instrument in running buffer consisting of HBS (20 mM HEPES, pH 7.5, 200 mM NaCl) and 1% DMSO. To determine the binding affinities, increasing concentrations of small molecules were injected onto the surface of the chip for 180 s at a flow rate of 30 μl/min, followed by a dissociation period of 330 s. Experimental data were collected and analyzed using Biacore Insight Evaluation software. The kinetic constant of binding was calculated using a 1:1 steady-state affinity model.

**Cell viability assay**

In total, 2 × 10^3^ of neutrophils were seeded in 96-well plates in triplicate in 100 µl of complete medium per well. After the indicated time points, 10 µl of Cell Counting Kit-8 (CCK-8) cell proliferation assay reagent (MedChemExpress) was added to the cells, and the cells were incubated at 37 °C for another 4 h. The optical absorbance was determined at 450 nm using a microplate reader (Thermo Fisher Scientific, Multiskan Go 1510).

**Supplementary Figures**


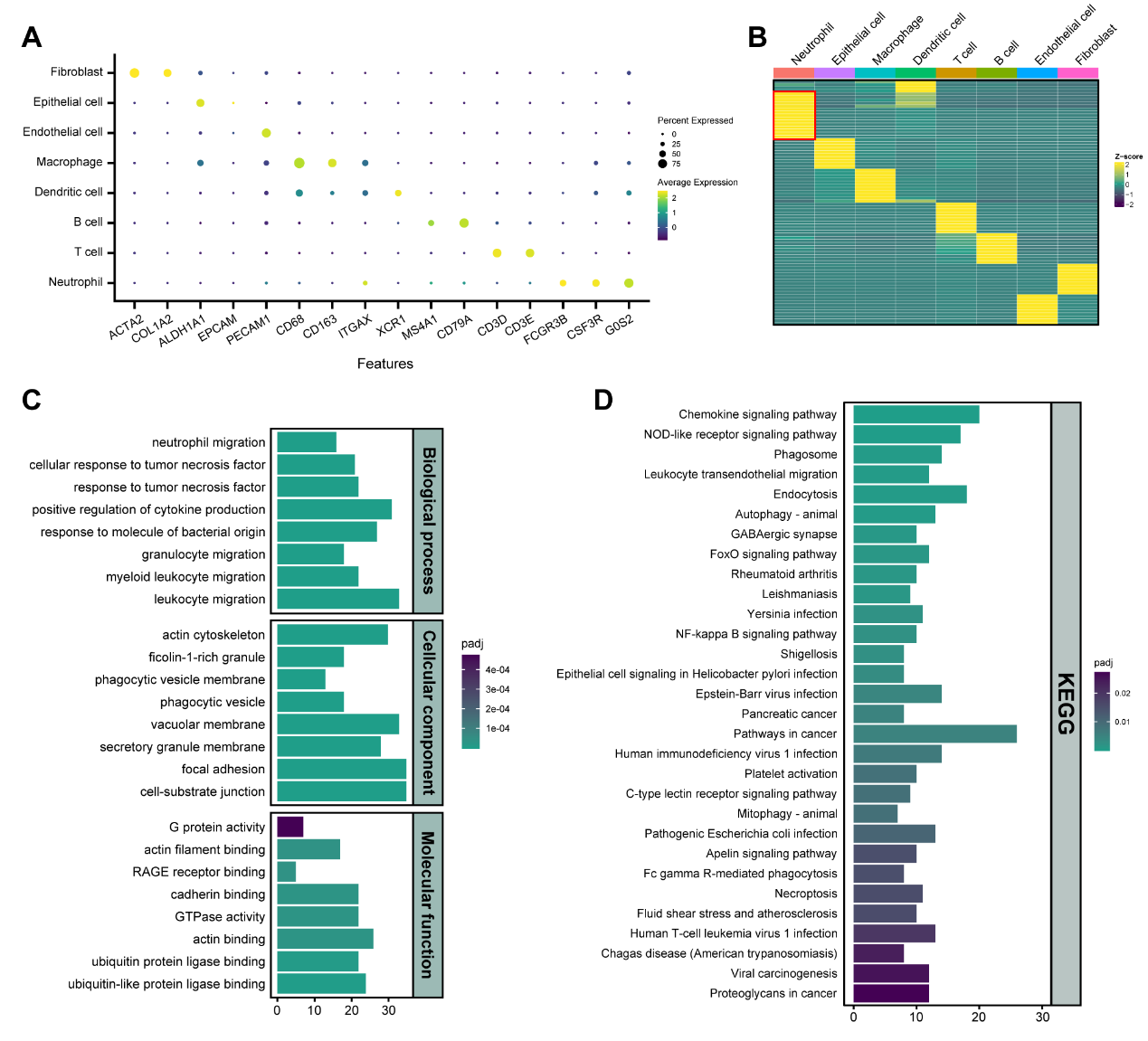
**Supplementary Figure S1. Single-cell RNA sequencing analyses identify neutrophil-specific marker genes in HCC.** (A) Bubble plots showing average expression of known markers in indicated cell clusters. (B) Heatmap of marker genes in each single cell subcluster based on the clustering analysis. (C) GO analysis of neutrophil maker genes. (D) KEGG analysis of neutrophil maker genes.


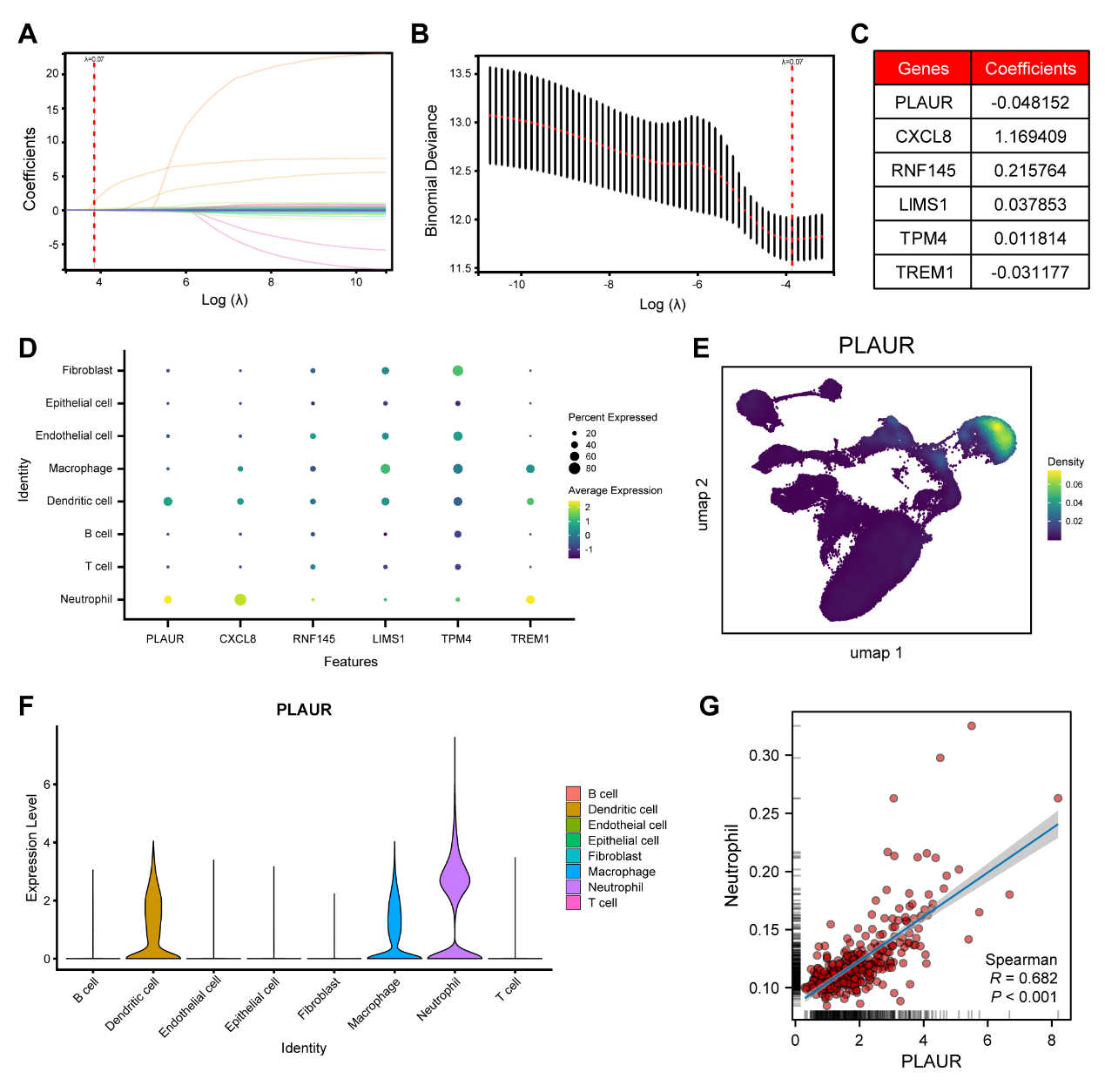


**Supplementary Figure S2. PLAUR is identified as a prognostic neutrophil-specific genes associated with immunotherapy response in HCC.** (A-C) LASSO analysis and 1000-fold cross-validation were used to identify the valuable immunotherapy-associated neutrophil-specific genes associated with prognosis in HCC. (D) Bubble plot of the expression levels of the candidate genes in each cell subcluster. (E) UMAP plot of PLAUR expression level in all clusters. (F) Violin plot of the PLAUR expression level in each cell subcluster. (G) Pearson correlation analysis of neoplastic PLAUR expression with the infiltration proportion of neutrophils.


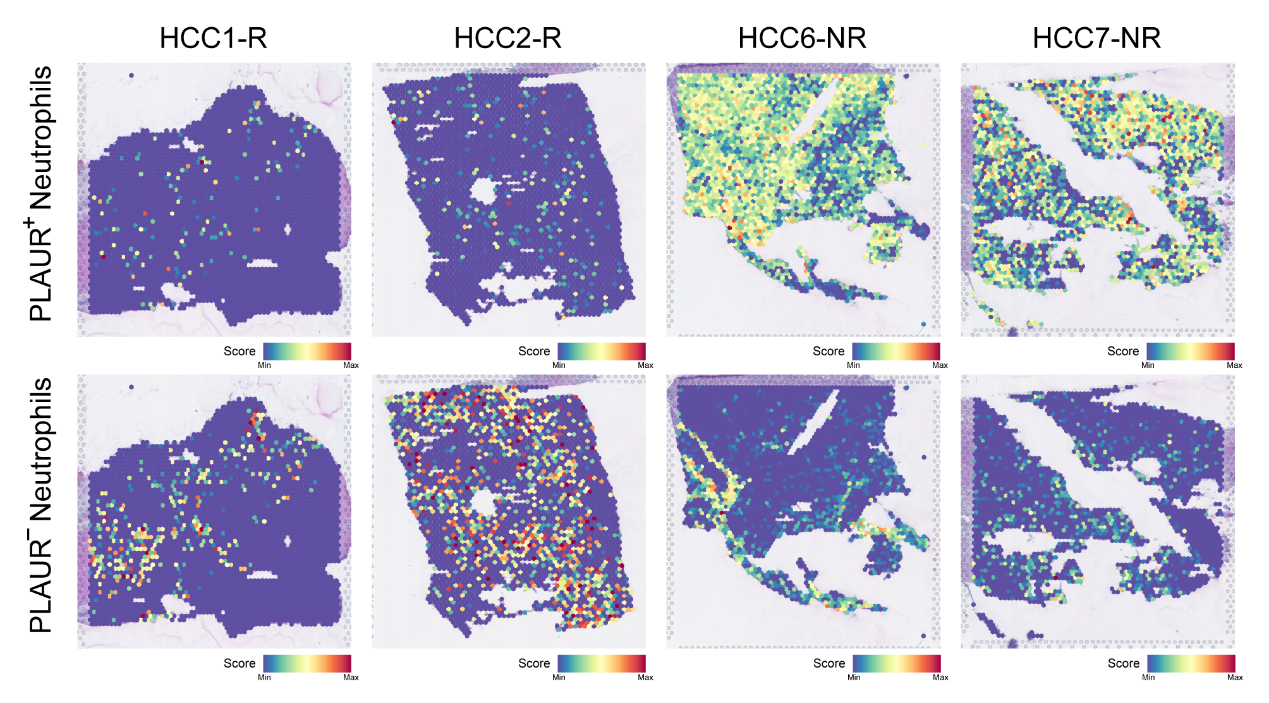


**Supplementary Figure S3. Distribution of PLAUR^+^/PLAUR^-^ neutrophils in HCC samples based on ST cohort (GSE238264).**

**
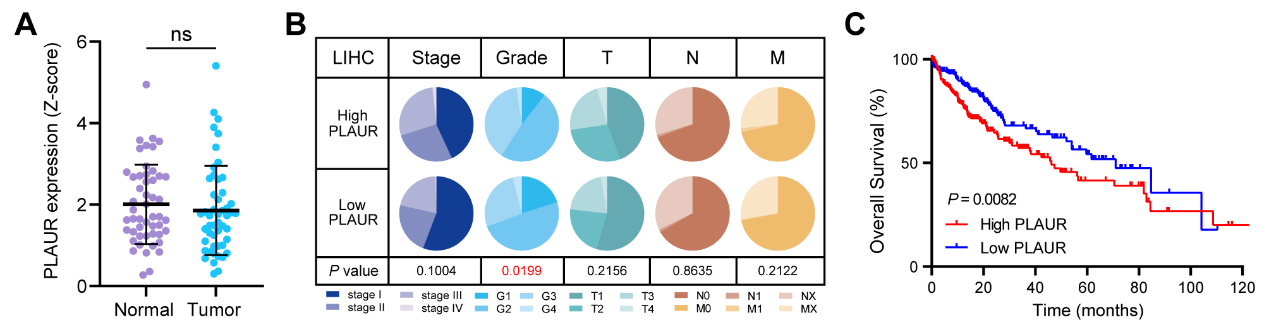
**

**Supplementary Figure S4. Clinical characteristics of the PLAUR high and low expression groups in the TCGA-LIHC cohort.** (A) Standardized transcript level of PLAUR in HCC tissues versus matched peritumor tissues in TCGA-LIHC cohort. (B) The circular pie chart shows the proportion difference of clinical indicators between the PLAUR high and low expression groups in the TCGA-LIHC cohort. (C) OS curves for patients in the PLAUR high and low expression groups. The data are presented as the means ± SDs. **P*<0.05, ***P*<0.01 and ****P*<0.001, Student’s *t* test.

**
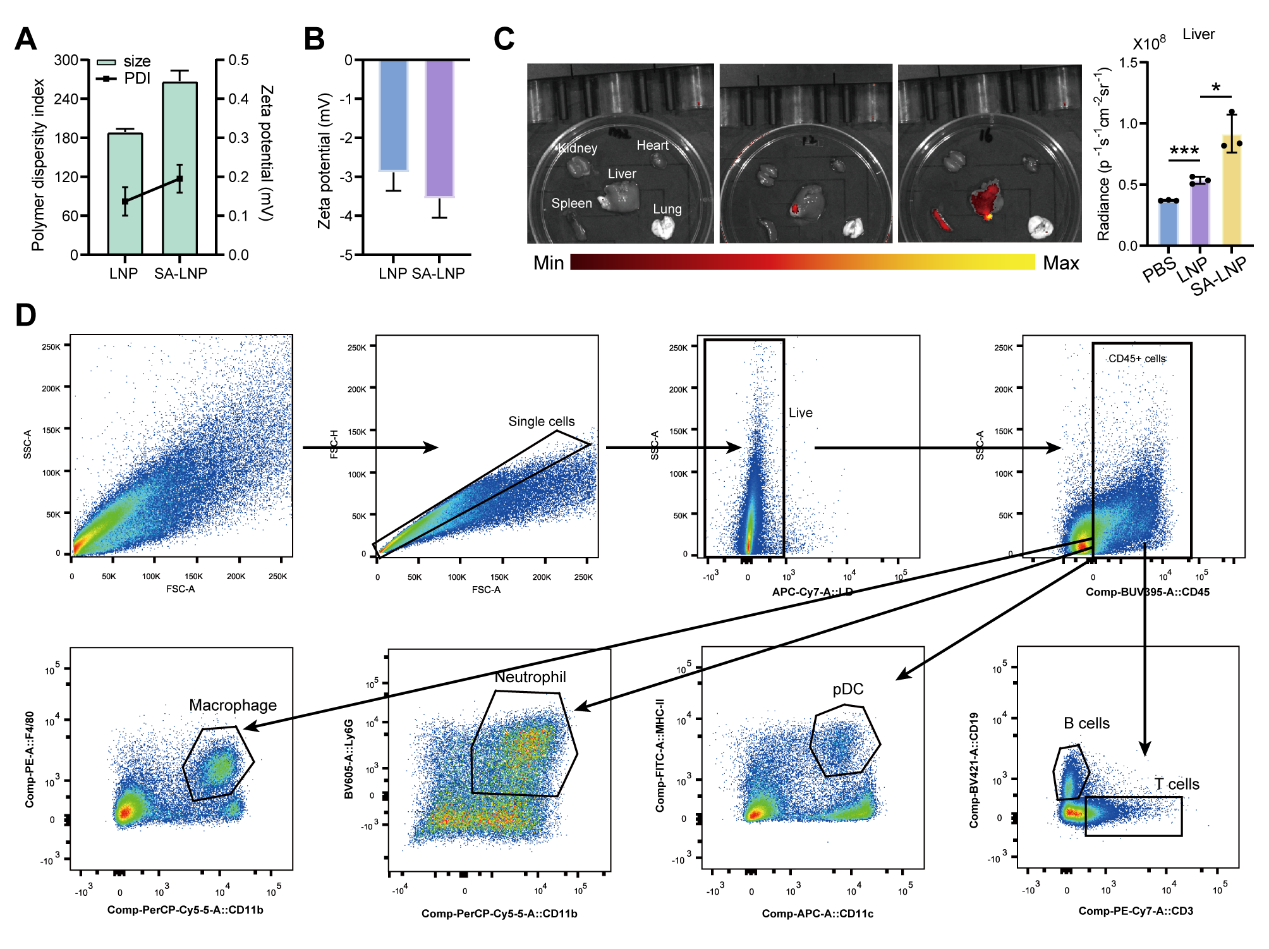
**

**Supplementary Figure S5. Characterization of a sialic acid-modified nanodelivery system (SA-LNPs).** (A, B) The particle size, PDI, and zeta potential of LNPs and SA-LNPs nanodelivery systems were determined by dynamic light scattering (n=3 per group). (C) Distribution and quantification of SA-LNPs in the major organs of tumor-bearing mice after intravenous injection (n=3 per group). (D) Gating strategy for cellular uptake of SA-LNPs-based mRNA vaccine delivery systems by hepatic cell types. The data are presented as the means ± SDs. **P*<0.05, ***P*<0.01 and ****P*<0.001, Student’s *t* test.


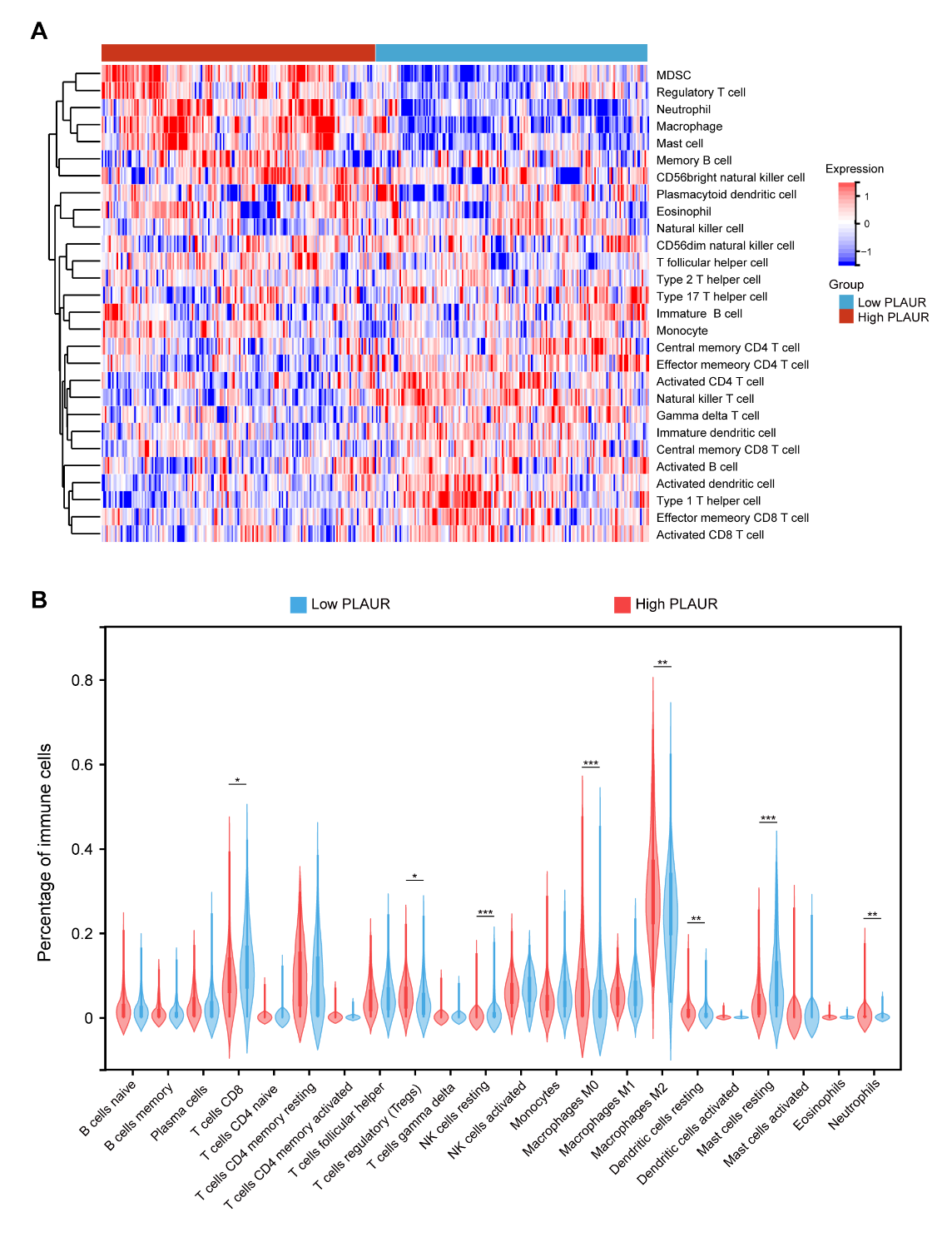


**Supplementary Figure S6. PLAUR expression correlates with immune exhaustion TME in HCC.** (A) Heatmap of immunological activity score calculated using ssGSEA in each tumor sample in TCGA-LIHC cohort based on PLAUR high and low expression groups. (E) Violin chart of the infiltration percentage of 22 immune cell types calculated using CIBERSORT algorithm in PLAUR high and low expression group. The data are presented as the means ± SDs. **P*<0.05, ***P*<0.01 and ****P*<0.001, Student’s *t* test.

**
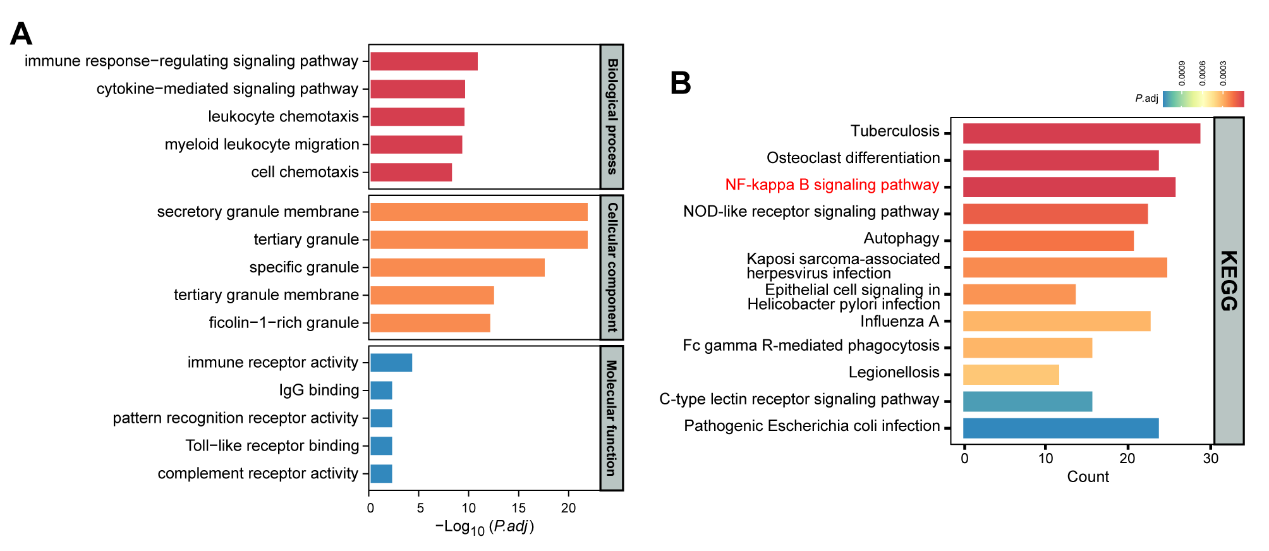
**

**Supplementary Figure S7. Functional enrichment analysis of differentially expressed genes between PLAUR^+^ and PLAUR^-^ neutrophils.** (A) GO analysis of differentially expressed genes between PLAUR^+^ and PLAUR^-^ neutrophils. (B) KEGG analysis of differentially expressed genes between PLAUR^+^ and PLAUR^-^ neutrophils.

**
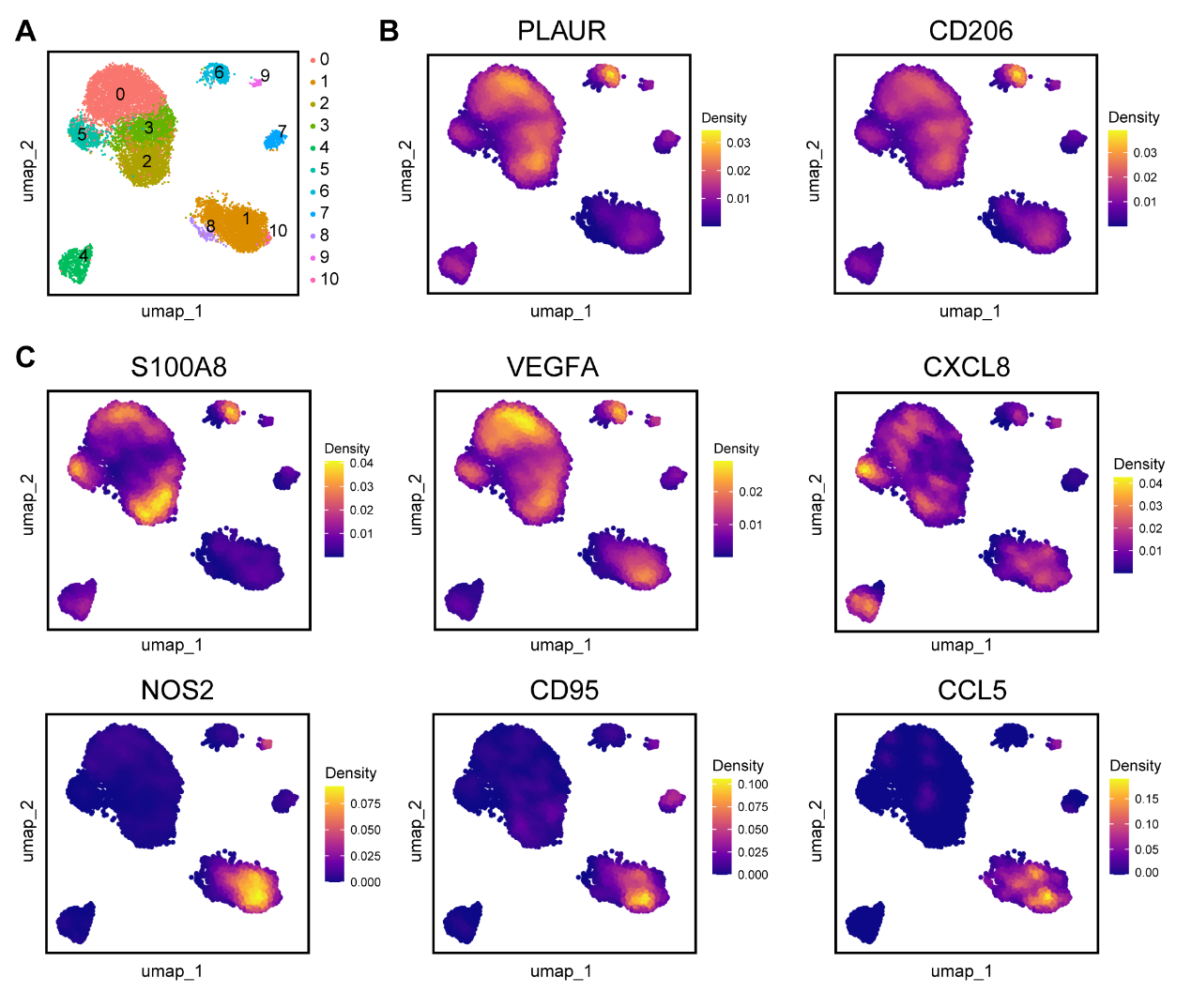
**

**Supplementary Figure S8. Single-cell RNA sequencing analyses of indicated genes expression in neutrophil subsets.** (A) UMAP plot of neutrophils clustering from PRJCA020880 and GSE202642 cohort. (B, C) UMAP plots of neutrophils clustering colored by indicated genes.

**
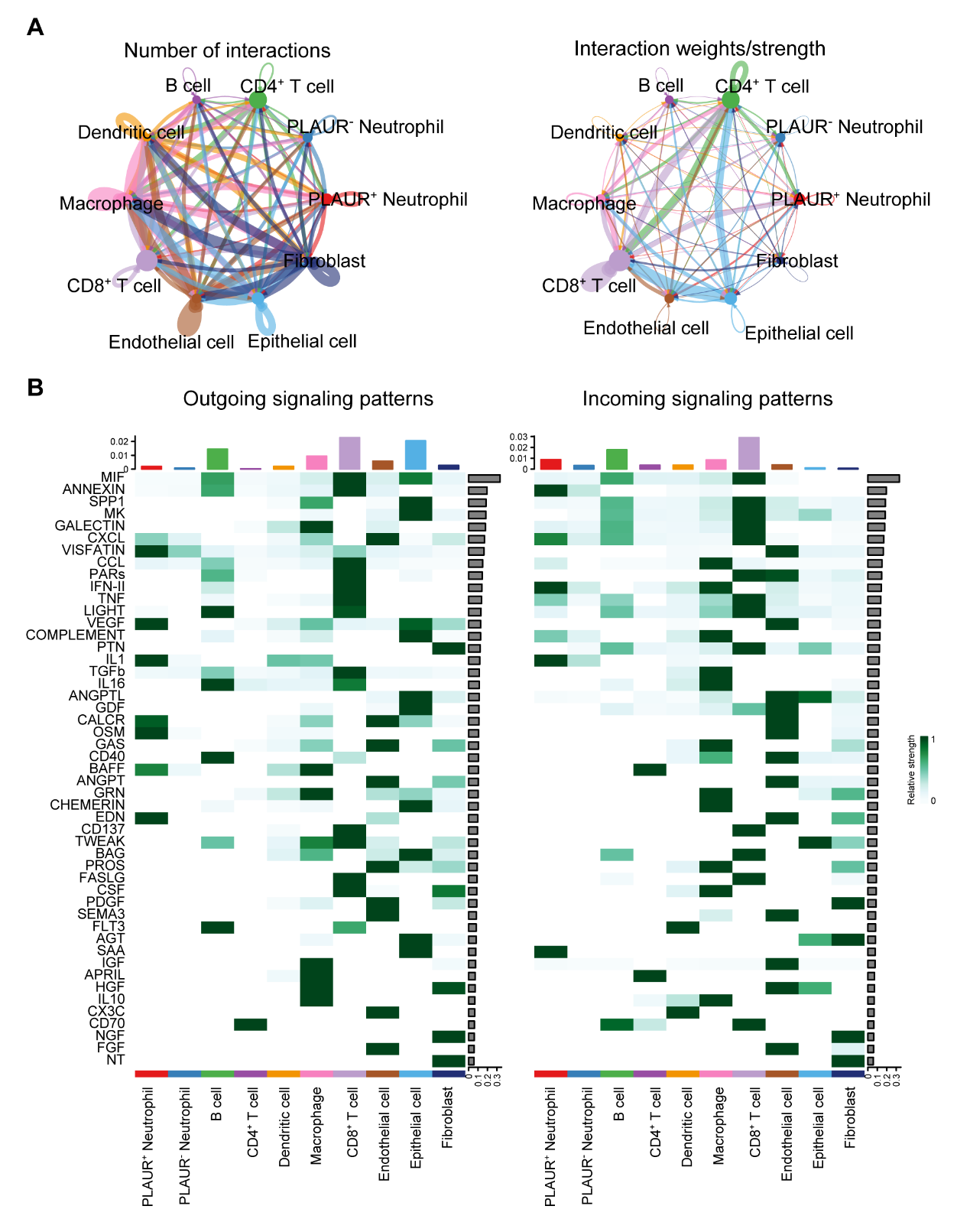
**

**Supplementary Figure S9. Inference of cell–cell communications in TME.** (A) Cell–cell communications between the identified cell types. (B) The outgoing and incoming signaling pathways of each cell type.

**
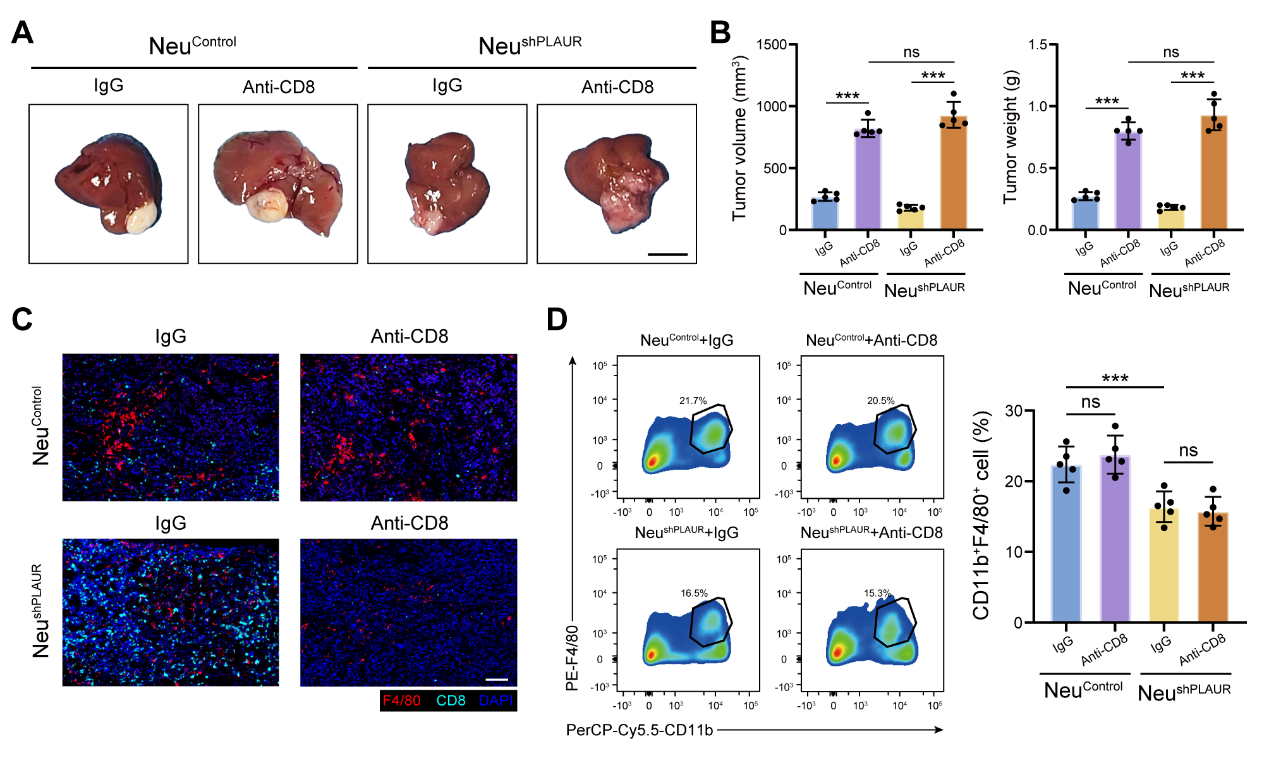
**

**Supplementary Figure S10. CD8 depletion abrogates antitumor effects of PLAUR knockdown neutrophil.** (A) Representative images of the orthotopic tumors at the study endpoint (5 mice per group). Scale bar: 1 cm. (B) The tumor volume and tumor weight of each group at the study endpoint (n=5 per group). (C) Immunofluorescence staining of F4/80 and CD8 in the indicated groups. Scale bars: 100 µm. (D) Flow cytometry analysis of CD11b^+^F4/80^+^ macrophages in the indicated groups (n=5 per group). The data are presented as the means ± SDs. **P*<0.05, ***P*<0.01, ****P*<0.001, One-way ANOVA with a post hoc LSD test.


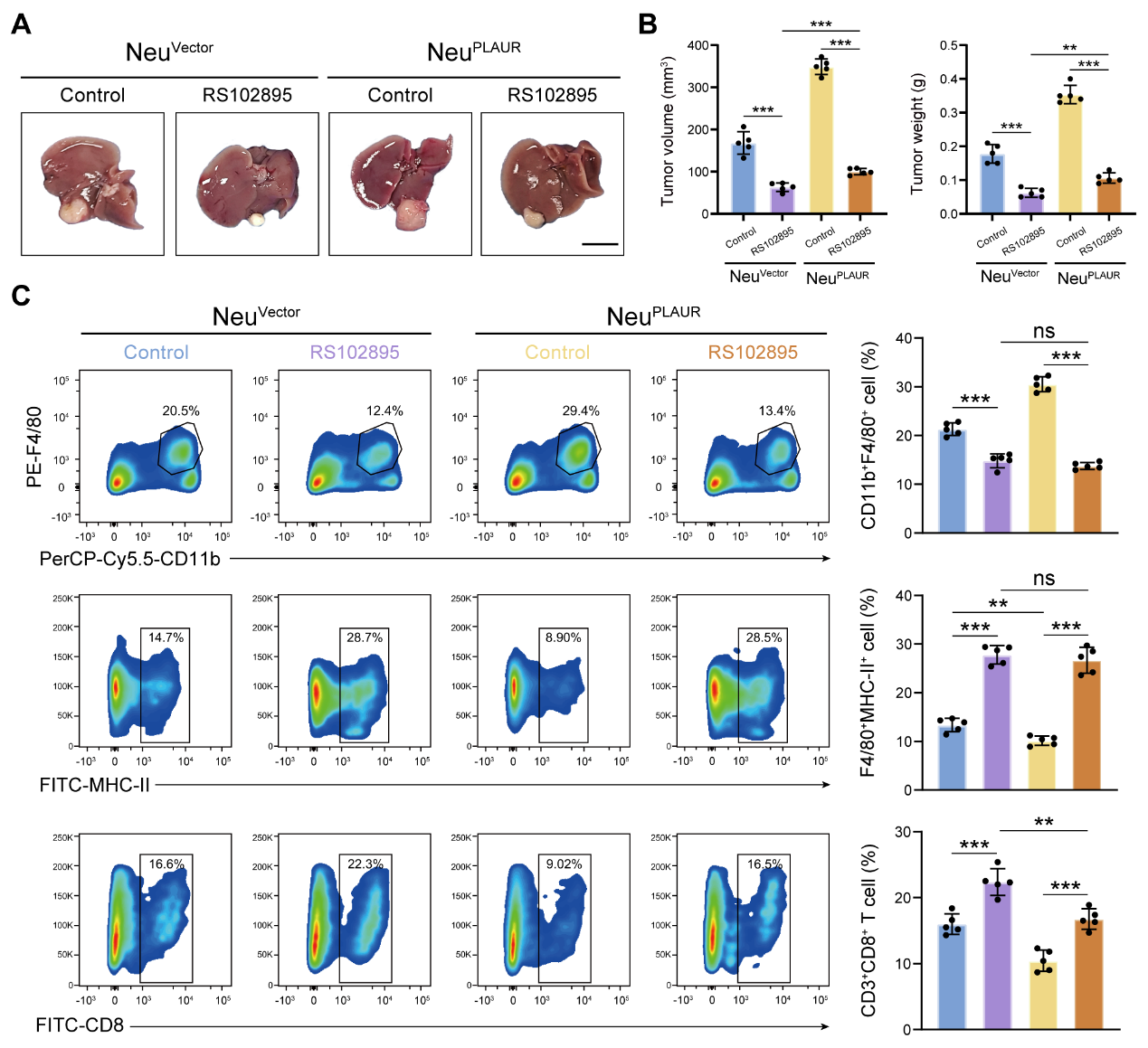


**Supplementary Figure S11. Blockade of CCL2-CCR2 axis** **significantly impaired PLAUR^+^ neutrophils-mediated HCC progression.** (A) Representative images of the orthotopic tumors at the study endpoint (5 mice per group). Scale bar: 1 cm. (B) The tumor volume and tumor weight of each group at the study endpoint (n=5 per group). (C) Flow cytometry analysis of CD11b^+^ F4/80^+^ macrophages, CD11b^+^ F4/80^+^ MHC-II^+^ macrophages and CD8^+^ T cells in each group (n=5 per group). The data are presented as the means ± SDs. ***P*<0.01, ****P*<0.001 and NS, not significant, One-way ANOVA with a post hoc LSD test.


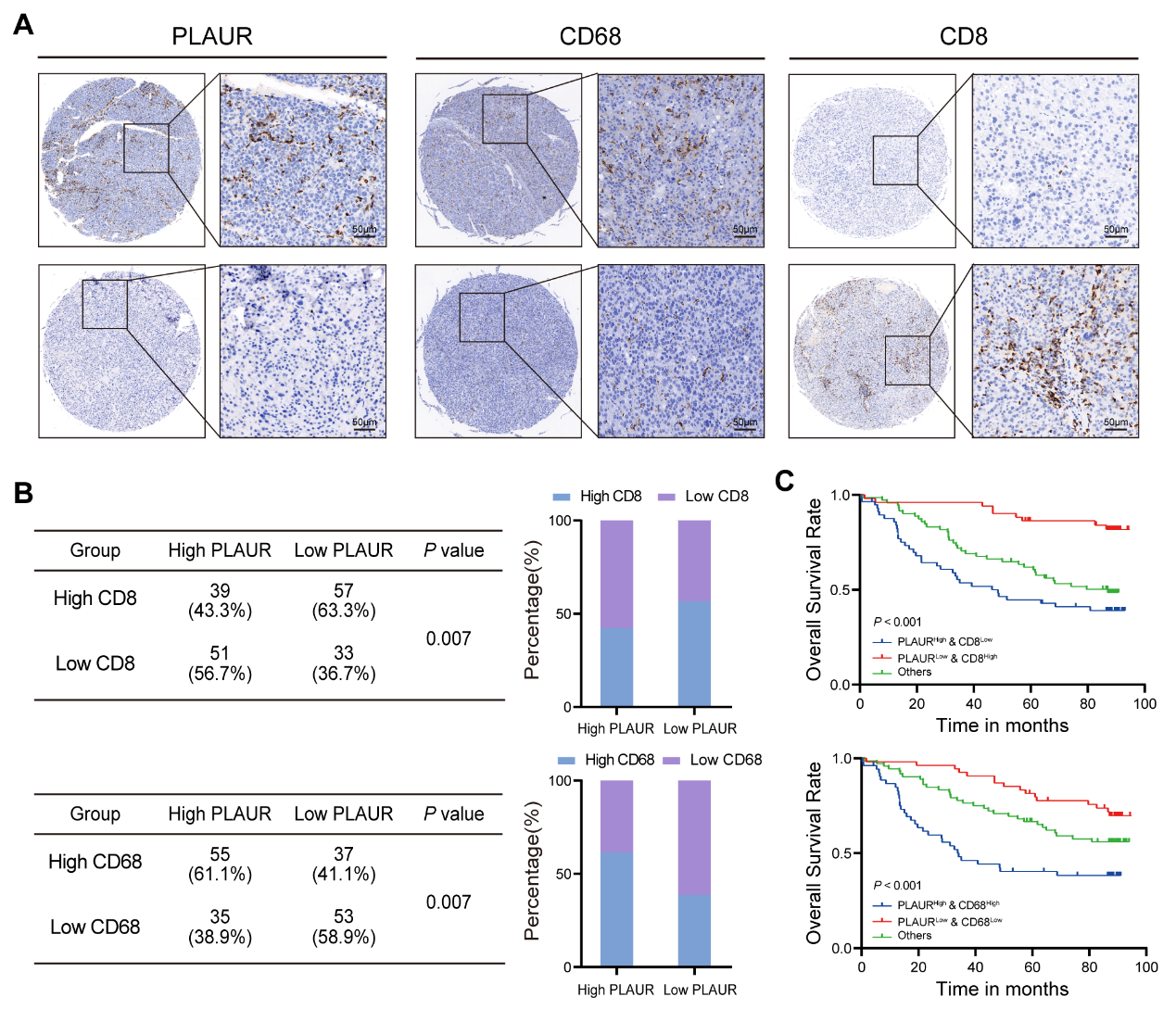


**Supplementary Figure S12. PLAUR expression correlates with CD8 and CD68 levels in HCC.** (A) Representative IHC staining images showing the expressions of PLAUR, CD68 and CD8 in our cohort 2. Scale bar: 50 μm. (B) Proportion of different PLAUR, CD68 and CD8 levels in our cohort 2. (C) OS curves for HCC patients with PLAUR/CD8 and PLAUR/CD68 co-expression. Pearson *χ*2 and Fisher’s exact test.


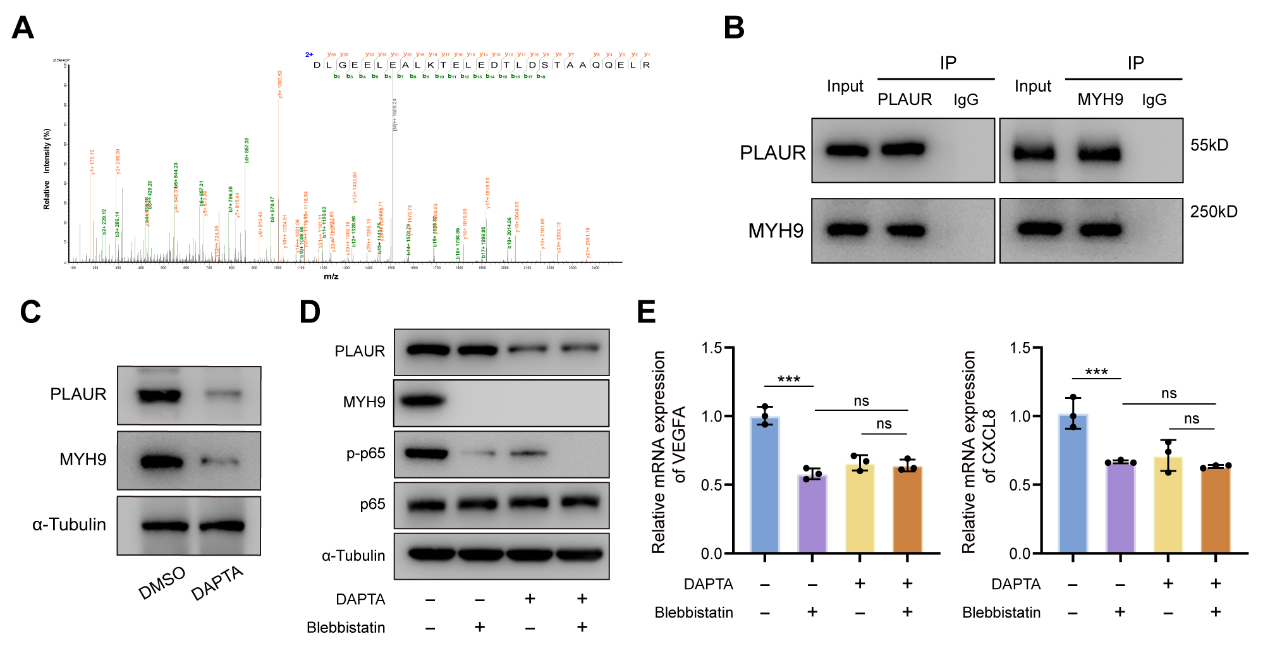


**Supplementary Figure S13. PLAUR activates NF-κB/p65 pathway through MYH9.** (A) The peptide spectrum of MYH9 determined by LC-MS/MS analysis in the immunoprecipitates of PLAUR. (B) IP analysis showing the interaction of PLAUR with MYH9 in neutrophils. (C) Western blot analysis of the protein level of MYH9 and PLAUR in the indicated neutrophils. (D) Western blot analysis of PLAUR, MYH9 and phosphorylated and non-phosphorylated p65 in the indicated neutrophils. (E) qRT-PCR analysis of VEGFA and CXCL8 transcriptional levels in the indicated neutrophils (n=3 per group). The data are presented as the means ± SDs. ****P*<0.001 and NS, not significant, One-way ANOVA with a post hoc LSD test.


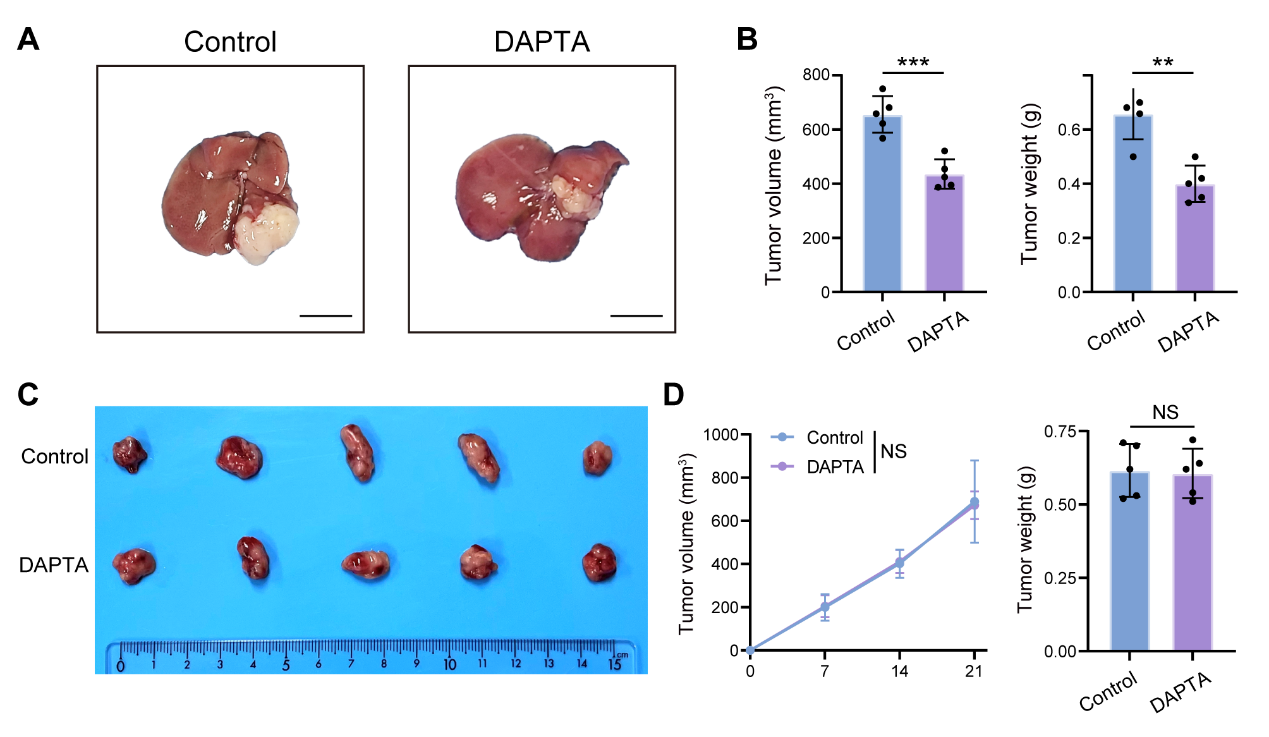


**Supplementary Figure S14. DAPTA exerts antitumor effects in an immune-dependent manner.** (A) Representative images of the orthotopic tumors at the study endpoint (5 mice per group). Scale bar: 1 cm. (B) The tumor volume and tumor weight of each group at the study endpoint (n=5 per group). (C) Gross appearance of the subcutaneous HCC tumors from immunodeficient mouse models in the indicated treatment groups (n=5 per group). (D)The tumor growth curves and weight of subcutaneous tumors in each group (n=5 per group). The data are presented as the means ± SDs. **P*<0.05, ***P*<0.01, and ****P*<0.001, Student’s *t* test.

| **Variables** | **Responder** | **Non-responder** | **P value** | |
| --- | --- | --- | --- | --- |
| Total | 17 | 25 |  | |
| Age(years) |  |  | 0.346 | |
| ≤55/>55 | 10/7 | 10/15 | |  |
| CA199 |  |  | | 0.309 |
| ≤34/>34U/L | 11/6 | 20/5 | |  |
| DBIL |  |  | | 0.754 |
| ≤6.8/>6.8ng/mL | 9/8 | 11/14 | |  |
| TBIL |  |  | | 0.208 |
| ≤20.4/>20.4ng/mL | 12/5 | 12/13 | |  |
| AFP |  |  | | 0.534 |
| ≤20/>20ng/mL | 9/8 | 16/9 | |  |
| Albumin |  |  | | 0.716 |
| ≤45/>45g/L | 14/3 | 19/6 | |  |
| GGT |  |  | | 0.757 |
| ≤60/>60U/L | 7/10 | 9/16 | |  |
| ALT |  |  | | 0.179 |
| ≤50/>50U/L | 14/3 | 15/10 | |  |
| AST |  |  | | 0.121 |
| ≤40/>40U/L | 8/9 | 18/7 | |  |
| Tumor size |  |  | | **<0.01** |
| ≤5/>5cm | 11/6 | 5/20 | |  |
| Cirrhosis |  |  | | 0.346 |
| No/yes | 7/10 | 15/10 | |  |
| BCLC |  |  | | 0.740 |
| A/B/C | 3/3/11 | 4/7/14 | |  |
| PVTT |  |  | | 0.530 |
| No/yes | 10/7 | 11/14 | |  |
| HBsAg |  |  | | 0.731 |
| Negative/Positive | 13/4 | 17/8 | |  |
| PLAUR^+^ neutrophil infiltration |  |  | | **<0.01** |
| Low/high | 12/5 | 6/19 | |  |

**Supplementary Table S1. Relationships between ICIs response and clinicopathological characteristics in the Cohort 1.**

**Supplementary Table S2. Details of 36 antibodies applied in CyTOF assay**

| **Label** | **Marker** | **Clone** | **Manufacturer** |
| --- | --- | --- | --- |
| 89Y | CD45 | HI30 | BioLegend |
| 115In | CD3 | UCHT1 | Bio Cell |
| 141Pr | CD86 | FUN-1 | BD |
| 142Nd | TCR γ/δ | 5A6.E9 | Thermo fisher |
| 143Nd | Granzyme B Recombinant | QA16A02 | BioLegend |
| 144Nd | CD28 | CD28.2 | BioLegend |
| 146Nd | CD123(IL-3Rα) | 6H6 | BioLegend |
| 147Sm | CD197(CCR7) | G043H7 | BioLegend |
| 148Nd | CD19 | HIB19 | BioLegend |
| 149Sm | CD169 | 7-239 | BioLegend |
| 150Nd | CD223(LAG-3) | 874501 | R&D |
| 152Sm | CD103 | B-Ly7 | eBioscience |
| 154Sm | CD163 | GHI/61 | BioLegend |
| 155Gd | CD45RA | HI100 | BioLegend |
| 156Gd | CD33 | WM53 | BioLegend |
| 157Gd | CD68 | Y1/82A | BioLegend |
| 158Gd | CD204(SR-AI) | 351615 | R&D |
| 159Tb | CD56 | NCAM16.2 | BD |
| 161Dy | CD152(CTLA-4) | 14D3 | eBioscience |
| 162Dy | CD25(IL-2Rα) | 24212 | R&D |
| 163Dy | CD278(ICOS) | C398.4A | BioLegend |
| 164Dy | CD64_plt | 10.1 | BioLegend |
| 165Ho | CD66b | G10F5 | BioLegend |
| 166Er | CD69 | FN50 | BioLegend |
| 167Er | CD206(MMR) | 15-2 | BioLegend |
| 168Er | CD11c | Bu15 | BioLegend |
| 170Er | CD39 | A1 | BioLegend |
| 171Yb | CD127(IL-7Rα) | A019D5 | BioLegend |
| 172Yb | CD279(PD-1) | EH12.2H7 | BioLegend |
| 173Yb | CD366(Tim-3) | F38-2E2 | BioLegend |
| 174Yb | CD14 | M5E2 | BioLegend |
| 175Lu | PLAUR | 3G8 | BioLegend |
| 176Yb | HLA-DR | L243 | BioLegend |
| 197Au | CD4 | RPA-T4 | BioLegend |
| 198Pt | CD8a | RPA-T8 | BioLegend |
| 209Bi | CD11b | M1/70 | BioLegend |

**Supplementary Table S3. Sequences of primers (5’-3’) used for qRT-PCR.**

| Genes | | Sequences (5’-3’) |
| --- | --- | --- |
| Human CD95 | FORWARD | GACCCTCCTACCTCTGGTTCTT |
| Human CD95 | REVERSE | CTGGAGGACAGGGCTTATGG |
| Human NOS2 | FORWARD | CGTGGAGACGGGAAAGAAGT |
| Human NOS2 | REVERSE | GACCCCAGGCAAGATTTGGA |
| Human CCL4 | FORWARD | CCCAGCCAGCTGTGGTATTC |
| Human CCL4 | REVERSE | CTCATGGAGAAGCATCCGGG |
| Human CCL5 | FORWARD | CCCATATTCCTCGGACACCAC |
| Human CCL5 | REVERSE | GCATCCTTGACCTGTGGACGA |
| Human TNF-α | FORWARD | TCTCCTTCCTGATCGTGGCA |
| Human TNF-α | REVERSE | CAGCTTGAGGGTTTGCTACAAC |
| Human CD206 | FORWARD | ATTCAGATATGCCAGGGCGA |
| Human CD206 | REVERSE | ATTTGGGTTCGGGAGTCGTC |
| Human S100A8 | FORWARD | ATGCCGTCTACAGGGATGAC |
| Human S100A8 | REVERSE | ACTGAGGACACTCGGTCTCTA |
| Human VEGFA | FORWARD | CAACAAATGTGAATGCAGACCAA |
| Human VEGFA | REVERSE | GCTCCAGGGCATTAGACAGC |
| Human CXCL8 | FORWARD | GAGAAGTTTTTGAAGAGGGCTGA |
| Human CXCL8 | REVERSE | CAACAGACCCACACAATACATGAAG |
| Human CXCR4 | FORWARD | CGATGGACTTCTAAACCAGCCA |
| Human CXCR4 | REVERSE | CCCACAATGCCAGTTAAGAAGA |
| Human MYH9 | FORWARD | AAGCTGGTATGGGTGCCTTC |
| Human MYH9 | REVERSE | CTTGGGCGGGTTCATCTTCT |
| Human GAPDH | FORWARD | CTGGGCTACACTGAGCACC |
| Human GAPDH | REVERSE | AAGTGGTCGTTGAGGGCAATG |

**Supplementary Table S4. Primary antibodies used in the study**

| Antibody | Concentration | Application | Identifier | Company |
| --- | --- | --- | --- | --- |
| NF-κB(p65) | 1:1000 | Western blot | Cat#8242 | Cell Signaling Technology |
| Phospho-NF-κB(p-p65) | 1:1000 | Western blot | Cat#3033 | Cell Signaling Technology |
| PLAUR | 1:200 | IHC | Cat#GTX100467 | GeneTex |
|  | 1:400 | IF |  |  |
|  | 1:1000 | Western blot |  |  |
| α-Tubulin | 1:1000 | Western blot | Cat#2125 | Cell Signaling Technology |
| CD66b | 1:100 | IF | Cat#ab229074 | Abcam |
| CD8a | 1:1000 | IHC | Cat#ab237709 | Abcam |
|  | 1:100 | IF |  |  |
| CD68 | 1:100 | IF | Cat#ab283654 | Abcam |
|  | 1:500 | IHC |  |  |
| F4/80 | 1:100 | IF | Cat#71299 | Cell Signaling Technology |
| Ly6G | 1:100 | IF | Cat#ab25377 | Abcam |
| PE anti-human CD66b | 5µl/million cells | Flow cytometry | Cat#392904 | Biolegend |
| PE/Cyanine7 anti-human CD16 | 5µl/million cells | Flow cytometry | Cat#980110 | Biolegend |
| APC anti-human CD206 (MMR) | 5µl/million cells | Flow cytometry | Cat#321110 | Biolegend |
| FITC anti-human CD95 (Fas) | 5µl/million cells | Flow cytometry | Cat#305606 | Biolegend |
| BUV395 Rat Anti-Mouse CD45 | 5µl/million cells | Flow cytometry | Cat#565967 | BD Bioscience |
| Zombie NIR™ Fixable Viability Kit | 5µl/million cells | Flow cytometry | Cat#565967 | Biolegend |
| PE/Cyanine7 anti-mouse CD3 | 5µl/million cells | Flow cytometry | Cat#100220 | Biolegend |
| Brilliant Violet 421™ anti-mouse CD19 | 5µl/million cells | Flow cytometry | Cat#152421 | Biolegend |
| FITC anti-mouse I-A/I-E | 5µl/million cells | Flow cytometry | Cat#107605 | Biolegend |
| APC anti-mouse CD11c | 5µl/million cells | Flow cytometry | Cat#117310 | Biolegend |
| PerCP/Cyanine5.5 anti-mouse/human CD11b | 5µl/million cells | Flow cytometry | Cat#101228 | Biolegend |
| Brilliant Violet 605™ anti-mouse Ly-6G | 5µl/million cells | Flow cytometry | Cat#127639 | Biolegend |
| PE anti-mouse F4/80 | 5µl/million cells | Flow cytometry | Cat#111604 | Biolegend |
| FITC anti-mouse CD8a | 5µl/million cells | Flow cytometry | Cat#100803 | Biolegend |
| PE/Cyanine7 anti-mouse CD8a | 5µl/million cells | Flow cytometry | Cat#100722 | Biolegend |
| APC anti-mouse CD206 (MMR) | 5µl/million cells | Flow cytometry | Cat#141708 | Biolegend |
| APC anti-mouse CD87 | 5µl/million cells | Flow cytometry | Cat#130128210 | Miltenyi Biotec |

**References**

1. Pan L, Zhang L, Deng W, Lou J, Gao X, Lou X*, et al.* Spleen-selective co-delivery of mRNA and TLR4 agonist-loaded LNPs for synergistic immunostimulation and Th1 immune responses. J Control Release **2023**;357:133-48

2. Wang F, Zhang M, Tian M, Lou J, Pan L, Gao X*, et al.* Natural long-chain saturated fatty acids doped LNPs enabling spleen selective mRNA translation and potent cancer immunotherapy. Nano Research **2024**;17:1804-17

3. Detre S, Saclani Jotti G, Dowsett M. A "quickscore" method for immunohistochemical semiquantitation: validation for oestrogen receptor in breast carcinomas. J Clin Pathol **1995**;48:876-8

4. Xu W, Weng J, Xu M, Zhou Q, Liu S, Hu Z*, et al.* Chemokine CCL21 determines immunotherapy response in hepatocellular carcinoma by affecting neutrophil polarization. Cancer Immunol Immunother **2024**;73:56
